# Care plans for women pregnant using assisted reproductive technologies: a systematic review

**DOI:** 10.1186/s12978-019-0667-z

**Published:** 2019-01-29

**Authors:** Maria P. Velez, Candyce Hamel, Brian Hutton, Laura Gaudet, Mark Walker, Micere Thuku, Kelly D. Cobey, Misty Pratt, Becky Skidmore, Graeme N. Smith

**Affiliations:** 1 Department of Obstetrics and Gynecology, Queen’s University, Kingston General Hospital, Kingston, Ontario K7L 2V7 Canada; 2 Department of Public Health Sciences, Queen’s University, 62 Fifth Field Company Lane, Kingston, Ontario K7L 3N6 Canada; 3 Knowledge Synthesis Group, Ottawa Methods Centre, Ottawa Hospital Research Institute, The Ottawa Hospital, 501 Smyth Road, Ottawa, Ontario K1H 8L6 Canada; 4 Department of Obstetrics, Gynecology & Newborn Care, University of Ottawa, 451 Smyth Road, Ottawa, Ontario K1H 8M5 Canada; 5 OMNI Research Group, Clinical Epidemiology Program, Ottawa Hospital Research Institute, 501 Smyth Road, Ottawa, Ontario K1H 8L6 Canada; 6 School of Epidemiology, Public Health and Preventive Medicine, University of Ottawa, 451 Smyth Road, Ottawa, Ontario K1H 8M5 Canada

**Keywords:** Systematic review, Assisted reproductive technologies, Pregnancy, Clinical practice guidelines, Antenatal care

## Abstract

**Background:**

Between 1 and 5% of children in industrialized countries are conceived through Assisted Reproductive Technologies (ART). As infertility and the use of ART may be associated with adverse perinatal outcomes, care plans specific to these pregnancies are needed. We conducted a systematic review to examine the existing care plans specific to women pregnant following Assisted Reproductive Technologies (ART).

**Methods:**

MEDLINE, Embase and the Cochrane Library were searched by a senior information specialist. The population of interest included women becoming pregnant with ART (e.g., Intra-Uterine Insemination, In Vitro Fertilization (IVF), Intracytoplasmic Sperm Injection (ICSI), and surrogacy). All proposed care plans were sought that pertained to any aspect of care during pregnancy and delivery. Only Clinical Practice Guidelines (CPGs) addressing the recommendations and plans for the care of ART pregnant women were included. The search was restricted to the publication dates 2007 to June 12, 2017 when the search was run. The search was not restricted by language, however only English and French language guidelines were considered for inclusion.

**Results:**

After screening 2078 citations, a total of ten CPGs were included. The following key clinical messages were prevalent: (1) although there was no supporting evidence, antenatal care for ART pregnancies should be provided by specialist with knowledge in obstetrics; (2) high-order multiple pregnancies are the greatest risk of ART and selective reduction options should be discussed; (3) there is some evidence of increased risk of congenital abnormalities and prenatal genetic and anatomic screening is recommended, especially in IVF-ICSI pregnancies; (4) due to a lack of or conflicting evidence, treatment of venous thromboembolism, antithrombotic therapy, treatment for hypothyroidism, and women with positive thyroid antibodies is recommended to be the same as in spontaneous pregnancies; and lastly (5) since an increased level of distress is a recognized feature in these pregnancies, psychosocial care and counselling should be considered.

**Conclusions:**

There is a lack of CPGs specific to ART pregnancies. While we identified a small number of recommendations for ART pregnancies, specific interventions and models of care aiming at decreasing adverse maternal and perinatal outcomes following ART should be developed, implemented, and evaluated.

**Electronic supplementary material:**

The online version of this article (10.1186/s12978-019-0667-z) contains supplementary material, which is available to authorized users.

## Plain English summary

Advances in infertility treatments have helped many couples to achieve a pregnancy. Some of these pregnancies may have a higher risk of complications for the mothers and the babies. The cause of infertility, the type of treatment, or both could play a role in these adverse events. Despite previous advances in the care of women who are expecting, there are few clinical practice guidelines specific to pregnant women who conceive with infertility treatment. Thus, we conducted a systematic review of current clinical practice guidelines to identify gaps in knowledge, including recommendations for clinical care and optimal maternity care provider and setting for women who conceived following infertility treatments. Only 10 guidelines were identified, and the quality of the evidence varied, with only one guideline considered of high quality. They recommend that antenatal care for these pregnancies should be provided by specialist with knowledge in obstetrics. In the case of a higher order multiple pregnancy, the parents should receive information about the risk/benefits of selective pregnancy reduction. Since some advanced infertility treatments may be associated with congenital abnormalities, prenatal genetic screening should be offered. The treatment of thromboembolic diseases and thyroid problems should be the same as for pregnancies conceived spontaneously. Finally, the stress associated with these pregnancies is recognized, and as such psychosocial support should be provided. We conclude that there is a lack of specific guidelines for pregnant women following infertility treatment, and new guidelines should be developed to decrease complications during pregnancy for this population.

## Background

Infertility has been declared as an emerging public health priority in developed countries [1]. It is estimated that 10–15% of couples experience infertility, which is defined as a failure to conceive after 12 months of unprotected intercourse [2]. Assisted reproductive technologies (ART) are used to assist couples attempting to overcome the challenge of infertility. Between 1 and 5% of children in industrialized countries are conceived through ART [3], and this number is expected to increase further as more countries provide access as part of their healthcare system [4].

Some studies suggest that ART pregnancies are associated with adverse maternal and perinatal outcomes, including preeclampsia, placenta previa, caesarean delivery, preterm birth, low birth weight, and congenital malformations, even among singleton pregnancies [5–7]. The reasons for this higher risk relate to both the underlying cause of infertility and the ART itself [8, 9]. Within the continuum of reproductive health care, antenatal care (ANC) aims to optimize maternal and perinatal outcomes through health promotion, screening and diagnosis, and disease prevention [10]. Currently, there are few clinical practice guidelines (CPGs) that address either the setting where ANC should be provided to pregnant women following ART or specific recommendations to be implemented with the aim to decrease adverse outcomes. Typically, couples are discharged from the fertility clinic to receive standard ANC, but there is currently little evidence to support whether this care adequately meets the need of ART pregnancies [11].

The objective of this systematic review was to identify the recommended care plans for women becoming pregnant with ART which are currently discussed in existing CPGs. This review focuses upon care given to women pregnant with the involvement of ART during pregnancy and delivery.

## Methods

This review has been reported with guidance from the PRISMA reporting guideline [12] and followed an a priori protocol, which was registered in PROSPERO (PROSPERO # CRD42017073228) and posted publicly in the University of Ottawa Library’s online repository (http://hdl.handle.net/10393/36555).

### Eligibility criteria

Criteria to identify eligible publications for the current review were established using the PICOS (Population-Intervention-Comparators-Outcomes-Study design) framework.

#### Population

The population of interest included women becoming pregnant with involvement of ART (e.g., Intra-Uterine Insemination (IUI), In Vitro Fertilization (IVF), Intracytoplasmic Sperm Injection (ICSI), and surrogacy).

#### Interventions/comparators/exposures

All proposed care plans were sought that pertained to any aspect of care for these women during pregnancy and delivery. This included women in both low risk (e.g., care from a general practitioner or midwife) and high risk settings (e.g., care from an obstetrician or maternal-fetal medicine).

#### Areas of interest

Any CPGs addressing the recommendations and plans for the care of ART pregnant women were included. Information of interest included the following: recommendations related to different types of maternity care providers (e.g., obstetrician, general practitioner, and midwife) and location of care (e.g., clinic, hospital); details of the care plans and/or individual elements recommended (e.g., including (but not limited to) the number and types of ultrasounds during pregnancy, prenatal screening, and so forth); underlying evidence supporting the recommendation (e.g., systematic reviews; if available, details on the approach to generate evidence for recommendations); citations of studies cited as informing the recommended care plans.

#### Study design

Only CPGs were included. Primary studies, abstracts, letters, commentaries, and non-guideline reviews were excluded. There were no restrictions imposed on the setting, or geographic location. The search was not restricted by language, however only English and French language guidelines were included.

The search strategies were developed and tested through an iterative process by an experienced medical information specialist (BS) in consultation with the review team. The strategies were peer reviewed by another senior information specialist prior to execution using the PRESS Checklist [13]. Using the OVID platform, we searched Ovid MEDLINE®, including Epub Ahead of Print and In-Process & Other Non-Indexed Citations, and Embase. We also undertook a grey literature search of guideline registries listed in CADTH’s *Grey Matters: a practical tool for search health-related grey literature* (https://www.cadth.ca/resources/finding-evidence/grey-matters) and targeted specialty societies. All searches were undertaken on June 12, 2017.

Strategies utilized a combination of controlled vocabulary (e.g., “Prenatal Care”, “Reproductive Techniques, Assisted”, “Clinical Protocols”) and keywords (e.g., “antenatal”, “ART”, “pathway”). A guidelines/care pathway filter was applied and vocabulary and syntax were adjusted across the two databases. The search was restricted to the publication dates 2007 to the present. Animal-only, opinion pieces and conference abstracts were removed from the results.

Specific details regarding the strategies appear in Additional file 1.

### Data collection and analyses

#### Study selection

Search results were de-duplicated in Reference Manager [14] before uploading to Distiller Systematic Review Software® [15]. Screening was performed in two stages: title/abstract screening and full text screening. Screening questions were developed and pilot-tested on a subset of records before implementation (50 references for title and abstract screening and 10 for full-text screening). All titles and abstracts were screened in duplicate by two independent reviewers, using the liberal accelerated method [16]. This method requires only one reviewer to assess an abstract as eligible for full text screening, and requires two reviewers to deem the abstract irrelevant. Full text articles for references included based on title and/or abstract were retrieved and assessed for inclusion at full-text screening, by two independent reviewers. Discrepancies were resolved by consensus. The process of study selection is reported below using a Preferred Reporting Items for Systematic Reviews and Meta-Analyses (PRISMA) flow diagram [12], including reasons for excluding full-text articles.

References that did not contain an abstract were screened based on the title, and those determined to be clearly not relevant were excluded. If there was any indication that the title may be relevant, or it was unclear, it was passed through to full-text screening

### Data collection

Data extraction forms were developed in Microsoft Excel 2007 and pilot tested on one included guideline. One reviewer extracted all data and a second reviewer verified all of the information collected. For all included CPGs, the following study characteristics were extracted: authorship list; guideline funders and sponsoring society; CPG type (new versus update of an existing CPG); date of publication; journal of publication/website; and country/language of publication. Other guideline information that were extracted included the following clinical details: recommendations related to different types of maternity care providers (e.g., obstetricians, general practitioners, midwife), location of care (e.g., clinic, hospital); details of the care plans and/or individual elements recommended (e.g., prenatal screening); underlying evidence (e.g., systematic reviews; if available, details on the approach to generate evidence for recommendations was collected); citations of studies cited as informing the recommended care plans.

### Quality assessment

Quality assessment was performed on each clinical research guideline using the Appraisal of Guidelines for Research & Evaluation (AGREE)-II tool [17]. This tool consists of six domains as follows: (1) scope and purpose, which addresses the overall objectives of the guideline, if the health questions are specifically described and if the population to whom the guideline is meant to apply is well described; (2) stakeholder involvement, which addresses who was involved in the development of the guideline, if the views and preferences of the target population have been sought, and if the target users are clearly defined; (3) rigor of development, which addresses the methodological quality of the guideline, including clear reporting of the criteria for inclusion, the strengths and limitations of the evidence, the methods for formulating the recommendations, external review of the guideline and a process for updating; (4) clarity of presentation, which addresses how well the recommendations are presented; (5) applicability, which addresses how well the guideline provides guidance on the implementation, barriers and facilitators to its application; and (6) editorial independence, which addresses the how the source of funding may have influence the content and any competing interests of the guideline development group.

For the current review, the checklist was implemented in DSR, and three reviewers independently assessed the quality of each included guideline using this checklist. Three reviewers were used to increase the validity of the overall findings, as suggested by the AGREE-II Next Steps Consortium [17]. For any question where there was a difference greater than two points in the assessment between all reviewers, the discrepancy was discussed and consensus was reached. Each domain score was calculated as described in the AGREE-II user’s manual [17]. As the Consortium has not set minimum domain scores or patterns of scores across domains to differentiate between high quality or poor quality guidelines, an overall quality score of 1–3 was considered low quality. An overall quality score of 4–5 was considered moderate quality. Lastly, a score of 6–7 was considered high quality. A narrative description of the quality assessment findings is presented, identifying domains of highest and lowest scores amongst the CPGs included in the review.

### Evidence syntheses

Recommendations from the included CPGs were summarized narratively according to the category of recommendations, which were chosen to be (1) models of care; (2) risks of ART; (3) surveillance, screening, and diagnostic testing in pregnancy; (4) treating conditions in pregnancy; and lastly (5) psychosocial care and counseling. Within each of these categories, we summarized recommendations described by at least one of the included CPGs, summarized the number of CPGs stating these recommendations, and indicated the assigned grade of evidence (where available). Within each of the categories of care, recommendations related to sub-aspects of care were grouped and contrasted where differences in recommendations were identified.

## Results

### Search results

**Fig. 1:**
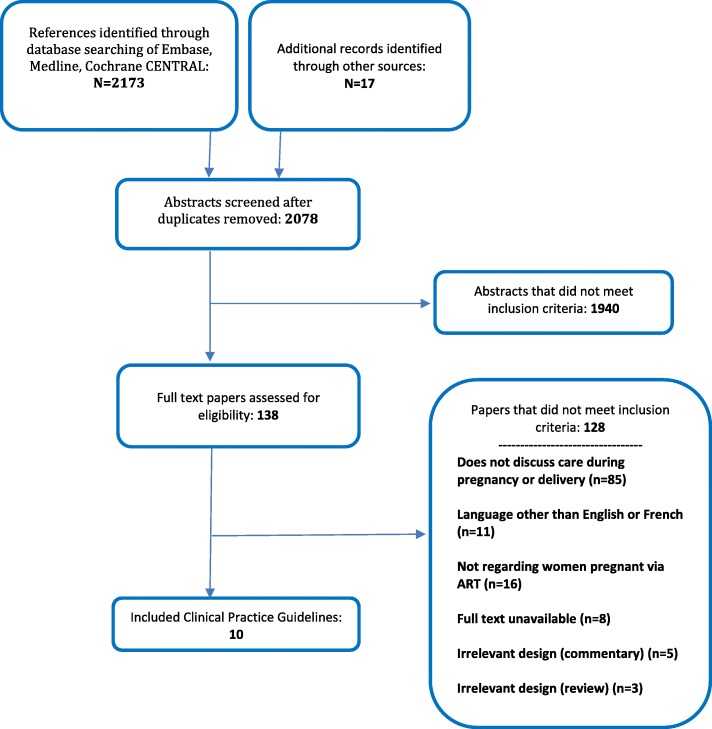
Flow diagram of study selection

The search across databases produced a total of 2173 records. After de-duplication and adding the records identified from the grey literature search and bibliographic screening of the included guidelines, 2078 unique records were assessed based on title and abstract. A total of 138 records were evaluated at full-text, and 10 guidelines were included (Fig. 1). Table 1 provides an overview of the primary characteristics of the included CPGs. [18–27]. Additional file 2 provides a list of studies that were excluded during full-text screening, with reasons.

**Table 1:** Characteristics of included CPGs

Guideline Characteristics						
Author/ Sponsoring Society/Title	Year	Country of Origin	Funding	Methods used	Update to previous guideline?	Grading of evidence performed?
Alexander et al. [23] Guidelines of the American Thyroid Association for the Diagnosis and Management of Thyroid Disease During Pregnancy and the Postpartum	2017	USA	American Thyroid Association (ATA)	- All task force members were provided written and verbal group advice on conducting electronic literature searches, critical appraisal of articles, and rationale for formulating strength of recommendations. - Standardized data collection forms were used by all reviewers. - For each question, a primary reviewer performed a literature search, appraised relevant literature, and generated recommendations, accompanying text, and a relevant bibliography. This information was then reviewed by both chairs, revised as needed, and presented for review by the entire panel. - Feedback and suggestions for revisions from the Chairs and panel members were obtained via e-mail, regularly scheduled teleconferences, and face-to-face meetings. - Once the manuscript was drafted, all suggestions for revisions were regularly reviewed by the entire panel in the form of a tracked changes draft manuscript and teleconferences. - The draft document continued to be revised until no suggestions for further revisions were requested by any panel members.	Updated from 2011	Yes, but no reference to any source.
American College of Obstetricians and Gynecologists [25]. *Perinatal Risks Associated With Assisted Reproductive Technology*	2016	USA	None stated	“based on available data and expert opinion”	Replaces Committee Opinion No. 324, November 2005	Not reported
American Society for Reproductive Medicine [24]. Subclinical *hypothyroidism in the infertile female population: a guideline*	2015	USA	None stated	- Systematic literature search using a combination of keywords restricted to MEDLINE citations of human subject research - Published in the English language from 1966 to March 2014 - Studies were eligible if they met one of the following criteria: primary evidence (clinical trials), that assessed the effectiveness of a procedure correlated with outcome measure (pregnancy, implantation, or live-birthrates), meta-analyses, and relevant articles from bibliographies of identified articles.	New	Using the American College of Physicians Grading System
Bates et al. [27]. *Venous thromboembolism, thrombophilia, Antithrombotic* *Therapy, and Pregnancy*	2012	Canada (based on first author). Produced for the American College of Chest Physicians	National Heart, Lung, and Blood Institute; Bayer Schering Pharma AG; Bristol-Myers Squibb; Pfizer, Inc.; Canyon Pharmaceuticals; and Sanofi Aventis US	- Literature search (January 2005–January 2010) - Published in English - Observational studies of pregnant women, including case reports and case series of pregnant women in the absence of studies with a cohort design	Updated from Antithrombotic and Thrombolytic Therapy: American College of Chest Physicians Evidence-Based Clinical Practice Guidelines (8th Edition)	GRADE (Grades of Recommendations, Assessment, Development, and Evaluation)
Chan et al. [26]. *Venous thromboembolism and antithrombotic therapy in pregnancy*	2014	Canada	None stated	- PubMed, Medline, CINAHL, and the Cochrane Library from Nov 2011 to Jul 2013 - Study designs restricted to systematic reviews, randomized control trials/controlled clinical trials, and observational studies - Published in English or French - There were no date restrictions - Grey literature was identified through searching several websites	Replaces No. 95, September 2000)	Using the ranking of the Canadian Task Force on Preventive Health Care
Chitayat et al. [19]. *Prenatal screening for fetal aneuploidy in singleton pregnancies*	2011	Canada	None stated	- PubMed or Medline and CINAHL and the Cochrane Library 1982 and 2009 - Study designs restricted to systematic reviews, randomized controlled trials, and relevant observational studies - No language restrictions - Searches were updated and incorporated to August 2010 - Grey literature was identified through searching several websites	Replaces No. 187, February 2007	Using the ranking of the Canadian Task Force on Preventive Health Care
Gameiro et al. [21]. *ESHRE guideline: routine psychosocial care in infertility and medically assisted reproduction-a guide for fertility staff*	2015	Europe	European Society of Human Reproduction and Embryology	- PUBMED, the Cochrane library, PsychInfo, and Embase published between Jan 1990 and Apr 2014 - Expert solicitation - Papers, dissertations, and book chapters were included, but conference abstracts were excluded - Literature searches were not limited to the English language - Literature searches were performed as an iterative process: (1) systematic reviews and meta-analyses; (2) randomized controlled trials; (3) prospective studies and case reports	New	Using the Scottish Intercollegiate Guidelines Network
Okun [18]. *Pregnancy outcomes after assisted human reproduction*	2014	Canada	None stated	- MEDLINE and the Cochrane Library from Jan 2005 to Dec 2012 - No restriction on study design - Published in English - Bibliography search of included articles - Searches were updated and incorporated to August 2013 - Grey literature was identified through searching several websites	Replaces No. 173, February 2006	Using the ranking of the Canadian Task Force on Preventive Health Care
The Royal Australian and New Zealand College of Obstetricians and Gynaecologists. [22]. *Maternal suitability for models of care, and indications for referral within and between models of care*	2015	Australia and New Zealand	The Royal Australian and New Zealand College of Obstetricians and Gynaecologists	- Declarations of interest were sought from all members prior to reviewing this statement - Structured clinical questions were developed and agreed upon - An updated literature search to answer the clinical questions was undertaken - The existing consensus-based recommendations were reviewed and updated (where appropriate) based on the available body of evidence and clinical expertise	Updated from March 2009	Not reported
Thorn and Wishmann [20]. *German guidelines for psychosocial counselling in the area of gamete donation*	2009	Germany	None stated	No systematic search procedure stated	New	Not reported

### Characteristics of included studies

Ten guidelines provided several recommendations for women who became pregnant using ART, although not all were specifically written to address only these pregnancies. Eight CPGs were published in 2012–2017 [18, 21–27] with two older CPGs published in 2009 [20] and 2011 [19]. Three CPGs were published from the Society of Obstetricians and Gynaecologists of Canada (SOGC), and all were updates that replaced older guidelines for the same topic. These guidelines focused on pregnancy outcomes after ART [18], venous thromboembolism and antithrombotic therapy in pregnancy [26], and prenatal screening for fetal aneuploidy in singleton pregnancies [19]. Two guidelines focused on psychosocial counseling specifically for gamete donation [20] and the fertility staff involved with medically assisted reproduction [21]. These were published by the German Infertility Counselling Network, and the European Society of Human Reproduction and Embryology (ESHRE), respectively. One guideline addressed maternal suitability for models of care from the Royal Australian and New Zealand College of Obstetricians and Gynaecologists (RANZCOG) [22]. One guideline addressed perinatal risks associated with ART from the American College of Obstetricians and Gynecologists (ACOG) [25]. Lastly, two CPGs addressed care to women during pregnancy with thyroid disorders [23, 24].

Six CPGs did not state how the development of the guideline was funded [18–20, 24–26]. The four CPGs that provided funding information were funded by the ESHRE [21], the RANZCOG [22], the American Thyroid Association [23], and the American College of Chest Physicians (ACCP) [27]. A total of five (50%) clearly indicated that recommendations were based upon systematic reviews of the evidence, and seven (70%) assigned formal grading of the recommendations; three CPGs [18, 19, 26] citing the Canadian Task Force on Preventive Health Care [28], one CPG [21] citing the Scottish Intercollegiate Guidelines Network [29], one CPG [27] citing Grading of Recommendations, Assessment, Development, and Evaluation (GRADE) [30], and one CPG [23] citing the American College of Physicians Grading System [31]. One CPG [24] used and described a grading system, but did not formally reference the system.

### Quality of the guidelines

Overall, the quality of the published guidelines varied, with three CPGs considered low quality, six considered moderate quality and one CPG developed from the ESHRE considered high quality [21], as it provided a link to the full CPG [32], which was assessed. Questions typically did not score well due to a lack of reporting, either by complete omission of the information, or from not including enough of the criteria as suggested by the AGREE-II user’s manual. A narrative description of each domain is provided next, while overall scoring of each domain is provided in Table 2.

**Table 2:** Summary of AGREE-II results

Guideline Identifiers		Aspects of AGREE-II Evaluation						
		Domains^a^						
Author	Year	Scope and purpose (%)	Stakeholder involvement (%)	Rigour of development (%)	Clarity and presentation (%)	Applicability (%)	Editorial independence (%)	Overall quality^b^
Alexander et al. [23]	2017	81	43	59	89	6	50	5 (moderate)
ACOG [25]	2016	22	26	17	57	6	0	2 (low)
ASRM [24]	2015	43	15	51	81	7	33	4 (moderate)
Bates et al. [27]	2012	74	19	36	78	10	89	4 (moderate)
Chan et al. [26]	2014	78	26	35	74	6	3	4 (moderate)
Chitayat et al. [19]	2011	81	43	44	83	17	3	5 (moderate)
Gameiro et al. [21]	2015	100	87	85	89	57	97	6 (high)
Okun and Sierra [18]	2014	70	41	46	78	7	6	4 (moderate)
RANZCOG [22]	2015	43	13	9	17	11	3	2 (low)
Thorne and Wischmann [20]	2009	46	13	3	69	7	0	2 (low)

^a^domain % scores were calculated using the methods described in the AGREE-II user’s manual

^b^overall quality scores were on a scale from 1 to 7, with 7 rating the highest quality. An overall quality score of 1–3 was judged as low quality. An overall quality score of 4–5 was judged as moderate quality. An overall quality score of 6–7 was judged as high quality

*ACOG* American College of Obstetricians and Gynecologists

*ASRM* American Society for Reproductive Medicine

*RANZCOG* The Royal Australian and New Zealand College of Obstetricians and Gynaecologists

Regarding the scope and purpose domain, scores ranged from 22 to 100% (median 72%). CPGs that scored low in this domain did not provide sufficient information on the target population of interest or a clear description of the health questions covered by the guideline.

With respect to the stakeholder involvement domain, scores ranged from 13 to 87% (median 29%). Guidelines typically specified the names and geographic location of the development group, but failed to specify discipline or content expertise, their institution, or a description of the member’s role in the guideline development group. Only one guideline [32] specifically sought the views and preferences of the target population and incorporated these perspectives into the guideline and its recommendations.

Concerning the rigour of development domain, scores ranged from 3 to 85% (median 40%). Excluding the CPG by the ESHRE, the range was 3 to 59%. In general CPGs were associated with a lack of reporting of several key details including (i) the methodology used for study selection; (ii) the methods used to formulate recommendations; (iii) the approach to how the external review was performed; and (iv) a description of the procedure for updating the guideline. None of the included CPGs provided the literature search strategies used or a link to the search strategies used.

In regard to the clarity of presentation domain, scores ranged from 17 to 89% (median 78%). One guideline received a low score [22]; after excluding it, scores ranged from 57 to 89%. Recommendations were specific, easily understood and identifiable, either by bullet points, numbered, or presented in greyed-out boxes.

With respect to the applicability domain, scores ranged from 6 to 57% (median 7%; range 6 to 17% without the ESHRE guideline) [21]. Overall, there was little or no information on advice on how to use the guideline in practice, the facilitators and barriers to its application, the potential resource implications of applying the recommendations, and the monitoring or auditing criteria of the CPG.

With reference to the editorial independence domain, scores ranged from 0 to 97% (median 4%). Funding information was not provided in most CPGs, and aside from generic statements that “disclosure statements have been received from all contributors”, there was no further information about any competing interests of the CPG development group members.

### Results of included guidelines

Narrative summaries with table-based presentations (Tables 1 and 3) are provided to summarize proposed aspects of care plans identified from the included literature. A detailed table of all recommendations and supporting publications is presented in Table 4. Most CPGs included any method of ART and may have provided information for a sub-type of ART (e.g., IVF-ICSI), while some were specific to a certain method (e.g., only donor insemination). CPG recommendations were grouped in five categories depending on the focus of the CPG: (1) models of care; (2) risks of ART; (3) surveillance, screening and diagnostic testing during pregnancy; (4) treating conditions in pregnancy; and (5) psychosocial counselling for those involved in ART.

**Table 3:** Summary of recommendations

	Alexander [23]	ACOG [25]	ASRM [24]	Bates [27]	Chan [26]	Chitayat [19]	Gameiro [21]	Okun [18]	RANZCOG [22]	Thorn [20]
Models of care										
Level of care recommended									✓	
Risks of ART										
Multi-fetal reduction options		✓								
Surveillance, screening and diagnostic testing in pregnancy										
Closer surveillance								✓		
U/S screening for congenital abnormalities		✓						✓		
Diagnostic testing for IVF-ICSI						✓		✓		
Labs should be aware of ART pregnancy						✓				
Treating conditions during pregnancy										
Treatment for VTE				✓	✓					
Treatment for thyroid disease	✓		✓							
Psychosocial care and counselling										
When individuals should be referred or offered counselling							✓			✓

*ART* Assisted Reproductive Technologies

*U/S* ultrasound

*IVF-ICSI* In Vitro Fertilization-Intracytoplasmic Sperm Injection

*VTE* Venous Thromboembolism

*ACOG* American College of Obstetricians and Gynecologists

*ASRM* American Society for Reproductive Medicine

*RANZCOG* The Royal Australian and New Zealand College of Obstetricians and Gynaecologists

**Table 4:** Detailed recommendations and supporting publications

Guideline reference	Population and Recommendations specific to care for women during pregnancy and delivery who became pregnant using ART		
Author Year	General ART-pregnancy related recommendations	List of studies cited as informing the recommendation^a^	Level of evidence (e.g. II-2A)
Alexander 2017 [23]	Recommendation 24: In women who achieve pregnancy following controlled ovarian hyperstimulation, TSH elevations should be treated according to the recommendations for pregnant women in general (Section VII Hypothyroidism and Pregnancy), as outlined below:	Ref 246: Poppe K. Thyroid function after controlled ovarian hyperstimulation in women with and without the hyperstimulation syndrome. *Fertil Steril* 2011; 96:241–245. Ref 247: Mintziori G. Thyroid function during ovarian stimulation: a systematic review. *Fertil Steril* 2011; 96:780–785. Ref 248: Muller AF. Decrease of free thyroxine levels after controlled ovarian hyperstimulation. *J Clin Endocrinol Metab* 2000; 85:545–548. Ref 249: Poppe K. Impact of ovarian hyperstimulation on thyroid function in women with and without thyroid autoimmunity. *J Clin Endocrinol Metab* 2004; 89:3808–3812. Ref 250: Poppe K. Thyroid function after assisted reproductive technology in women free of thyroid disease. *Fertil Steril* 2005; 83:1753–1757. Ref 251: Gracia CR. Thyroid function during controlled ovarian hyperstimulation as part of in vitro fertilization. *Fertil Steril* 2012; 97:585–591. Ref 252: Reinblatt S. Thyroid stimulating hormone levels rise after assisted reproductive technology. *J Assist Reprod Genet* 2013; 30:1347–1352. Ref 255: Stuckey BG. Thyroxine replacement during super-ovulation for in vitro fertilization: a potential gap in management? *Fertil Steril* 2010; 93:2414.e1–3. Ref 256: Busnelli A. Thyroid axis dysregulation during in vitro fertilization in hypothyroid-treated patients. *Thyroid* 2014; 24:1650–1655. Ref 257: Busnelli A. Levothyroxine dose adjustment in hypothyroid women achieving pregnancy through IVF. *Eur J Endocrinol* 2015; 173:417–424. Ref 258: Davis LB. The effect of infertility medication on thyroid function in hypothyroid women who conceive. *Thyroid* 2007; 17:773–777.	Weak recommendation, moderate-quality evidence
	Recommendation 25: In the setting of pregnancy, maternal hypothyroidism is defined as a TSH concentration elevated beyond the upper limit of the pregnancy-specific reference range.	Ref 17: Li C et al. Assessment of thyroid function during first-trimester pregnancy: what is the rational upper limit of serum TSH during the first trimester in Chinese pregnant women? J Clin Endocrinol Metab 2014; 99:73–79. Ref 19: Korevaar TI, Hypothyroxinemia and TPO-antibody positivity are risk factors for premature delivery: the generation R study. J Clin Endocrinol Metab 2013; 98:4382–4390 Ref 24 Bestwick JP et al. Thyroid stimulating hormone and free thyroxine in pregnancy: expressing concentrations as multiples of the median (MoMs). Clin Chim Acta 2014; 430:33–37. Ref 265: La’ulu SL, Roberts WL. Ethnic differences in first trimester thyroid reference intervals. Clin Chem 2011; 57:913–915. Ref 266: Mannisto T et al. Early pregnancy reference intervals of thyroid hormone concentrations in a thyroid antibody-negative pregnant population. Thyroid 2011; 21:291–298. Ref 267: Medici M, et al. Maternal early pregnancy and newborn thyroid hormone parameters: the Generation R study. J Clin Endocrinol Metab 2011; 97:646–652. Ref 268. Springer D et al. Reference intervals in evaluation of maternal thyroid function during the first trimester of pregnancy. Eur J Endocrinol 2009; 160:791–797. Ref 269: Medici M, et al. Thyroid function in pregnancy: what is normal? Clin Chem 2015 61:704–713.	Strong recommendation, high-quality evidence
	Recommendation 26: The pregnancy-specific TSH reference range should be defined as follows: a) When available, population- and trimester-specific reference ranges for serum TSH during pregnancy should be defined by a provider’s institute or laboratory and should represent the typical population for whom care is provided. Reference ranges should be defined in healthy TPOAb-negative pregnant women with optimal iodine intake and without thyroid illness. When this goal is not feasible, pregnancy-specific TSH reference ranges obtained from similar patient populations and performed using similar TSH assays should be substituted. (Strong recommendation, high-quality evidence) b) If internal or transferable pregnancy-specific TSH reference ranges are not available, an upper reference limit of *4.0 mU/L may be used. For most assays, this limit represents a reduction in the nonpregnant TSH upper reference limit of *0.5 mU/L		a) Strong recommendation, high-quality evidence b) Strong recommendation, high-quality evidence c) Strong recommendation, moderate-quality evidence
American College of Obstetricians & Gynecologists 2016 [25]	Recommendation: When a higher-order (triplet or more) multifetal pregnancy is encountered, the option of multifetal reduction should be discussed. In the case of a continuing higher-order multifetal pregnancy, ongoing obstetric care should be with an obstetrician–gynecologist or other obstetric care provider and at a facility capable of managing anticipated risks and outcomes.	Ref 9: American College of Obstetricians and Gynecologists. ACOG Committee opinion no. 553: multifetal pregnancy reduction. Obstet Gynecol. 2013;121(2 Pt 1):405-410.(33) Ref 27: Wimalasundera RC. Selective reduction and termination of multiple pregnancies. *Semin Fetal Neonatal Med* 2010; 15:327-335. Ref 30: Dodd JM. Reduction of the number of fetuses for women with a multiple pregnancy. *Cochrane Database of Systematic Reviews* 2015, Issue 11. Art. No.: CD003932. pub3	Not stated
	"When a patient request for multifetal pregnancy reduction is discordant with the physician's value system, the patient should be referred to a physician with expertise in performing multifetal pregnancy reductions."	Ref 9: American College of Obstetricians and Gynecologists. ACOG Committee opinion no. 553: multifetal pregnancy reduction. Obstet Gynecol. 2013;121(2 Pt 1):405-410.(33)	Not stated
	"…, it seems judicious to make patients aware of the low level risk of birth defects and to offer ultrasonographic surveillance for structural abnormalities in these pregnancies. Some professional organizations recommend fetal echocardiography in all ART pregnancies, but the incremental yield of such studies after a targeted ultrasonography that is reassuring is unclear and needs to be balanced against available resources. Of course, patient-specific risks identified during evaluation of a patient's medical history may indicate need for specific studies or other fetal evaluation during pregnancy."	Ref 56: American Institute of Ultrasound in Medicine, AIUM Practice Parameter for the performance of fetal echocardiography. Laurel (MD): AIUM; 2013. Available at: http://www.aium.org/resources/guidelines/fetalEcho.pdf. Ref 57: Donofrio MT. Diagnosis and treatment of fetal cardiac disease: a scientific statement from the American Heart Association. American Heart Association Adults with Congenital Heart Disease Joint Committee of the Council on Cardiovascular Disease in the Young and Council on Clinical Cardiology, Council on Cardiovascular Surgery and Anesthesia, and Council on Cardiovascular and Stroke Nursing. *Circulation* 2014; 129:2183-242.	Not stated
ASRM 2015 [24]	Recommendation: Levothyroxine treatment may improve pregnancy outcomes in women with positive thyroid antibodies, especially if the TSH level is over 2.5 mlU/L.	Ref 48: Kutteh WH Increased prevalence of antithyroid antibodies identified in women with recurrent pregnancy loss but not in women undergoing assisted reproduction. *Fertil Steril* 1999; 71: 843–8. Ref 49: Poppe K. Assisted reproduction and thyroid autoimmunity: an unfortunate combination? *J Clin Endocrinol Metab* 2003; 88: 4149–52. Ref 54: Singh A. Presence of thyroid antibodies in early reproductive failure: biochemical versus clinical pregnancies. *Fertil Steril* 1995; 63: 277–81. Ref 55: Negro R. Euthyroid women with autoimmune disease undergoing assisted reproduction technologies: the role of autoimmunity and thyroid function. *J Endocrinol Invest* 2007; 30:3–8. Ref 56: Negro R. Levothyroxine treatment in thyroid peroxidase antibody-positive women undergoing assisted reproduction technologies: a prospective study. *Hum Reprod* 2005; 20:1529–33	Not clear
Bates 2012 [27]	Recommendation 5.1.1: For women undergoing assisted reproduction, we recommend against the use of routine thrombosis prophylaxis.	Ref 116: Mára M. Thromboembolic complications in patients undergoing in vitro fertilization: retrospective clinical study [in Czech]. *Ceska Gynekol* 2004; 69 (4): 312 - 316. Ref 117: Aurousseau MH. Risk of thromboembolism in relation to an in-vitro fertilization programme: three case reports. *Hum Reprod* 1995; 10 (1): 94-97. Ref 121: Jacobsen AF. Ante- and postnatal risk factors of venous thrombosis: a hospital-based case-control study. *J Thromb Haemost* 2008; 6 (6): 905-912. Ref 136: Hull RD. Extended out-of hospital low-molecular-weight heparin prophylaxis against deep venous thrombosis in patients after elective hip arthroplasty: a systematic review. *Ann Intern Med* 2001; 135 (10): 858-869.	Grade 1B
	Recommendation 5.1.2: For women undergoing assisted reproduction who develop severe ovarian hyperstimulation syndrome, we suggest thrombosis prophylaxis (prophylactic LMWH) for 3 months postresolution of clinical ovarian hyperstimulation syndrome rather than no prophylaxis.	Ref 115: Nelson SM. Prophylaxis of VTE in women-during assisted reproductive techniques. *Thromb Res* 2009; 123 (suppl 3): S8-S15. Ref 116: Mára M. Thromboembolic complications in patients undergoing in vitro fertilization: retrospective clinical study [in Czech] *Ceska Gynekol* 2004; 69 (4): 312-316. Ref 122: Chan WS. T he ‘ART’ of thrombosis: a review of arterial and venous thrombosis in assisted reproductive technology. *Curr Opin Obstet Gynecol* 2009; 21 (3): 207-218.	Grade 2C
Chan 2014 [26]	Recommendation 41: Women who develop a venous thromboembolism in association with the use of assisted reproductive technology and conceive, follow recommendation 12 and 13.	see below	see below
	Recommendation 12: For pregnant women with an acute venous thromboembolism we recommend therapeutic anticoagulation for a minimum of 3 months.	" ... evidence confirming or disputing the safety of this option is unavailable" pg 535	I-A Unsure what they are basing this on
	Recommendation 13: Following initial treatment, anticoagulation intensity can be decreased to intermediate or prophylactic dose for the remainder of the pregnancy and for at least 6 weeks postpartum12 and 13 for acute venous thromboembolism in pregnancy.	"….evidence confirming or disputing the safety of this option is unavailable." pg 535	III-C Unsure what they are basing this on
	Recommendation 59 (b): Postpartum thromboprophylaxis should be considered in the presence of multiple clinical or pregnancy-related risk factors when the overall absolute risk is estimated to be greater than 1% : (b) in any 3 or more of the following risk factors (each with an absolute risk of venous thromboembolism < 1% in isolation): (i) age >35 years; (ii); parity ≥2; (iii) any assisted reproductive technology; (iv) multiple pregnancy; (v) placental abruption; (vi) premature rupture of membranes; (vii) elective Caesarean section; (viii) maternal cancer.	Ref 109: Jacobsen AF. Ante- and postnatal risk factors of venous thrombosis: a hospital-based case–control study. *J Thromb Haemost* 2008; 6:905–12.	II-2B
Chitayat 2011 [19]	Recommendation 4b: Invasive prenatal diagnosis for cytogenetic analysis should not be performed without multiple marker screening results except for women who are at increased risk of fetal aneuploidy because the pregnancy was conceived by in vitro fertilization with intracytoplasmic sperm injection.	Ref 4: Bonduelle M. Prenatal testing in ICSI pregnancies: incidence of chromosomal anomalies in 1586 karyotypes and relation to sperm parameters. *Hum Reprod* 2002; 17:2600–14.	II-2E
	Recommendation 13: Information such as gestational dating, maternal weight, ethnicity, insulin-dependent diabetes mellitus, and use of assisted reproduction technologies should be provided to the laboratory to improve accuracy of testing.	Ref 96: Barkai G. Down’s syndrome screening marker levels following assisted reproduction. *Prenat Diagn* 1996; 16:1111–4. Ref 97: Wald NJ. Serum markers for Down’s syndrome in women who have had in vitro fertilisation: implications for antenatal screening. *Br J Obstet Gynaecol* 1999; 106:1304–6. Ref 98: Perheentupa A. Maternal serum beta-HCG and alpha-fetoprotein concentrations in singleton pregnancies following assisted reproduction. *Hum Reprod* 2002; 17:794–7. Ref 99: Raty R. Serum free beta-HCG and alpha-fetoprotein levels in IVF, ICSI and frozen embryo transfer pregnancies in maternal mid-trimester serum screening for Down’s syndrome. *Hum Reprod* 2002; 17:481–4. Ref 100: Maymon R. Serial first- and second-trimester Down’s syndrome screening tests among IVF-versus naturally-conceived singletons. *Hum Reprod* 2002; 17:1081–5. Ref 101: Maymon R. Integrated first- and second-trimester Down syndrome screening test among unaffected IVF pregnancies. *Prenat Diagn* 2004; 24:125–9. Ref 102: Muller F. French Collaborative Group. Medically assisted reproduction and second trimester maternal serum marker screening for Down syndrome. *Prenat Diagn* 2003; 23:1073–6. Ref 103: Liao AW. First-trimester screening for trisomy 21 in singleton pregnancies achieved by assisted reproduction. *Hum Reprod* 2001; 16:1501–4. Ref 104: Orlandi F. First trimester screening with free beta-hCG, PAPP-A and nuchal translucency in pregnancies conceived with assisted reproduction. Prenat Diagn 2002; 22:718–21. Ref 105: Bellver J. First trimester biochemical screening for Down’s syndrome in singleton pregnancies conceived by assisted reproduction. *Hum Reprod* 2005; 20:2623–7. Ref 106: Hui PW. Nuchal translucency in pregnancies conceived after assisted reproduction technology. *Ultrasound Obstet Gynecol* 2005; 25:234–8.	II-2A
Gameiro 2015 [21]	Recommendation under section 4.3 (b): The guideline development group recommends that fertility staff refer patients who experience or are at risk of experiencing clinically significant psychosocial problems after successful treatment, to specialized psychosocial care (infertility counselling or psychotherapy).	Glade AC, Bean RA, Vira R. A Prime Time for Marital/Relational Intervention: A Review of the Transition to Parenthood Literature with Treatment Recommendations. *Am J Fam Ther* 2005;33: 319-336.	Good practice points based on expert opinion
	Recommendation under section 4.3 (b): The guideline development group recommends that fertility staff offer additional psychosocial care to patients at risk of increased infertility-specific psychosocial distress after successful treatment.	Hammarberg K, Fisher JR, Wynter KH. Psychological and social aspects of pregnancy, childbirth and early parenting after assisted conception: a systematic review. *Hum Reprod Update* 2008;14: 395-414	Good practice points based on expert opinion
	Recommendation under section 4.3 (b): The guideline development group recommends that fertility staff offer patients the opportunity to discuss their worries about pregnancy achieved with fertility treatment.	Ref 1: Vilska S. Mental health of mothers and fathers of twins conceived via assisted reproduction treatment: a 1-year prospective study. *Hum Reprod* 2009; 24: 367-377. Ref 2: Baor L. Mothers of IVF and spontaneously conceived twins: a comparison of prenatal maternal expectations, coping resources and maternal stress. *Hum Reprod* 2010; 25: 1490-1496.	Good practice points based on expert opinion
Okun 2014 [18]	Recommendation 6: There is a role for closer obstetric surveillance of women who conceive with assisted human reproduction	No references cited for this recommendation	III-L
	Recommendation 10: In pregnancies achieved by artificial reproductive technology, routine anatomic ultrasound for congenital structural abnormalities is recommended between 18 and 22 weeks.	Ref 28: Zhu JL. Infertility, infertility treatment, and congenital malformations: Danish national birth cohort. *BMJ* 2006; 333:679. Ref 38: Davies GA. Obesity in pregnancy. Society of Obstetricians and Gynaecologists of Canada Clinical Practice Guideline, No. 239, February 2010. *J Obstet Gynaecol Can* 2010; 32:165–73. Ref 149: Wennerholm UB. Incidence of congenital malformations in children born after ICSI. *Hum Repro* 2000; 15:944-8. Ref 150: Reefhuis J. Assisted reproductive technology and major structural birth defects in the United States. *Hum Reprod* 2009; 24:360–6. Ref 151: Wen SW. A comprehensive assessment of outcomes in pregnancies conceived by in vitro fertilization/ intracytoplasmic sperm injection. *Eur J Obstet Gynecol Reprod Biol* 2010; 150:160–5. Ref 152: Hansen M. The risk of major birth defects after intracytoplasmic sperm injection and in vitro fertilization. *N Engl J Med* 2002; 346:725-30. Ref 153: Katalinic A. Pregnancy course and outcome after intracytoplasmic sperm injection: a controlled, prospective cohort study. *Fertil Steril* 2004; 1:1604-16.	II-2A Some discrepancies between references in text and those in reference list.
	Recommendation 11: Pregnancies conceived by intracytoplasmic sperm injection may be at increased risk of chromosomal aberrations, including sex chromosome abnormalities. Diagnostic testing should be offered after appropriate counselling	Ref 149: Wennerholm UB. Incidence of congenital malformations in children born after ICSI. *Hum Reprod* 2000; 15:944–8. Ref 155: Bonduelle M. Prenatal testing in ICSI pregnancies: incidence of chromosomal anomalies in 1586 karyotypes and relation to sperm parameters. *Hum Reprod* 2002; 17:2600–14. Ref 156: Feng C. Assisted reproductive technology may increase clinical mutation detection in male offspring. I 2008; 90:92–6. Ref 158: Ranta JK. Increased time-to-pregnancy and first trimester Down's syndrome screening. *Hum Reprod* 2010: 25:412-7. Ref 159: Amore DJ. Pregnancies conceived using assisted reproductive technologies (ART) have low levels of pregnancy-associated plasma protein-A (PAPP-A) leading to a high rate of false-positive results in first trimester screening for Down syndrome. *Hum Reprod* 2009; 24:1330-8. Ref 160: Shulman LP. Maternal serum analyte levels after first-trimester multifetal pregnancy reduction. *Am J Obstet Gynecol* 1996; 174:1072-4. Ref 161: Shulman A. Mid-gestation Down syndrome screening test and pregnancy outcome among unstimulated assisted-conception pregnancies. *Prenat Diagn* 2003; 23:625-8.	II-2A Some discrepancies between references in text and those in reference list.
RANZCOG [22]	IVF or GIFT pregnancy should be referred to a GP (with a recognised postgraduate qualification in obstetrics) or Specialist Obstetrician where a GP with suitable qualifications is not available, referral should be to a specialist Obstetrician.	There are no references in this publication.	Unknown
Thorne and Wischmann [20]	Recommendation 3.6: During medical treatment and pregnancy, both partners may develop ambivalent feelings towards the fact that the female partner carries the semen of an unknown man or has become pregnant with this semen. Counselling can contribute towards an understanding to such reactions and help in managing them.	References were not linked to recommendations. A list of references are included in this publication, but other than the introduction section, none of contained within the remaining of the document.	None stated

^a^Only the first author is listed in the bibliographic reference

#### Models of care

One CPG reviewed the models of care in Australia and New Zealand, and referral within and between models [22]. This guideline is not specific to women who became pregnant using ART, but provides guidance on what type of clinician should care for women who needed IVF or gamete intrafallopian transfer (GIFT) to conceive. The recommendation is that these women should be followed by a general practitioner with a recognized postgraduate qualification in obstetrics. In the case where a GP with suitable qualifications is not available, referral should be to a specialist obstetrician.

#### Risks of ART

The ACOG guideline discussed the perinatal risks associated with ART [25]. Multi-fetal pregnancy (triplets or more) is more common in pregnancies achieved through ART, and its associated outcomes are the greatest risk of ART. This CPG provides a recommendation to discuss the risk and options if this occurs and makes reference to another CPG by the ACOG on multi-fetal pregnancy reductions and states: “when a patient request for multi-fetal pregnancy reduction is discordant with the physician’s value system, the patient should be referred to a physician with expertise in performing multi-fetal pregnancy reductions [33].”

#### Surveillance, screening, and diagnostic testing in pregnancy

##### Surveillance

Two SOGC guidelines discussed surveillance, screening and/or diagnostic testing for these women as there are several known risks (e.g., preeclampsia, preterm birth) associated with pregnancy achieved with ART [18, 19]. Although there was insufficient formal evidence, due to these known additional risks and/or other factors that may influence decision-making, expert opinion was considered in the recommendation that there is a need for closer surveillance during these pregnancies.

##### Screening

Several well-designed observational studies cited by the SOGC guidelines reported a higher prevalence of congenital malformations (Hazard Ratio (HR) 1.20; 95%CI 1.07–1.35), genital organ malformations (HR 2.32; 95%CI 1.24–4.35), and congenital defects, including septal heart defects (adjusted Odds Ratio (aOR) 2.1; 95%CI 1.1–4.0), esophageal atresia (aOR 4.5; 95%CI 1.9–10.5), and anorectal atresia (aOR 3.7; 95%CI 1.5–9.1) compared to spontaneously conceived infants [34, 35]. This suggests that an ultrasound for congenital abnormalities is recommended. The ACOG guideline also suggested that these women should be offered ultrasonographic surveillance for structural abnormalities and identified some professional organizations that recommend fetal echocardiography in all ART pregnancies, although the incremental yield is unclear [25].

Studies have evaluated several different maternal serum levels (e.g., Alfa fetoprotein-(AFT), Estradiol (uE3), Pregnancy associated plasma protein-A (PAPP-A), Human chorionic gonadotropin (HCG), etc) during the first and second trimesters of pregnancy. In observational studies cited by the CPG, there has been conflicting evidence that there are differences in some of these serum levels between pregnancies through IVF and non-IVF [36–38]. As screening programs typically collect information on IVF, it is recommended that this information is provided to the laboratory, but further investigation is necessary to determine if adjustment is necessary [19].

##### Diagnostic testing

Although the incidence of chromosomal abnormalities in births and induced terminations following IVF (0.7%) has been shown to be similar to those in spontaneous conceived pregnancies (0.2%), it has been shown to be significantly higher among those who became pregnant with IVF-ICSI (1.0%) [18]. Further supported by a SOGC guideline on prenatal screening for fetal aneuploidy in singleton pregnancies [19], in the case of pregnancy conceived by IVF-ICSI, the risk of chromosomal abnormality is high enough to offer invasive testing without prior non-invasive screening or based on a non-invasive screen result above the risk cut-off.

#### Treating conditions in pregnancy

The SOGC and ACCP CPGs focused on venous thromboembolism (VTE) and antithrombotic therapy in pregnancy and on how to diagnose and treat VTE in pregnancy and postpartum [26, 27]. One of the 67 recommendations in the SOGC CPG and two of the 37 recommendations from the ACCP CPG addressed women who became pregnant with ART and how to treat them during pregnancy. The SOGC CPG authors stated that the risk in women undergoing ART is estimated to be 0.11% per cycle of IVF (3 cases among 2748 IVF cycles) [39], similar to the general population of pregnant women (1 in 1000 pregnancies) [40]. The ACCP CPG found that while ART may be a risk factor, the incidence of thrombosis in ART patients was low (0.1 and 0.3%) [39, 41]. However, the risk of thrombosis was found to be higher in women with Ovarian Hyperstimulation Syndrome (OHSS), although based on observational data (up to 4.1% (95% CI, 1.1–13.7% in severe cases) [39]. There is little to guide clinicians in the use of thromboprophylaxis in women undergoing ART. Deriving from observational data of pregnant women (not specific to ART) at high risk for VTE (e.g. personal history of previous VTE, asymptomatic thrombophilia, family history of symptomatic thrombophilia, combined pregnancy-related risk factors) recommendations from the SOGC CPG are that thromboprophylaxis should be initiated if pregnancy is achieved. Among those with no risk factors for VTE, routine thromboprophylaxis is unnecessary. The ACCP CPG recommends against routine thromboprophylaxis for women undergoing ART. For those who develop severe OHSS, thromboprophylaxis for 3 months postresolution of the condition is suggested.

Two CPGs focused on women with thyroid disease, specifically hypothyroidism, and how it should be treated in pregnancy [23, 24]. Although both CPGs developed recommendations with the consideration of RCT and observational studies using women pregnant through ART (e.g., ovarian hyperstimulation, IVF), both CPGs recommend treating these women the same as those who conceived spontaneously. This is mainly due to the high level of conflicting evidence in these studies. Specific recommendations focus on treating TSH elevations and offering levothyroxine treatment to improve pregnancy outcomes in women with positive thyroid antibodies.

#### Psychosocial care and counselling

A guideline from the ESHRE [21] provided information for all fertility clinic staff (e.g., doctors, nurses, midwives, counselors, social workers) on when they should refer patients for additional psychosocial care after a successful pregnancy with ART treatment. No interventions were found to address behavioural, relational and social, emotional, and cognitive needs of these patients. The reviewed evidence suggested that the needs of couples who achieved pregnancy with fertility treatment did not differ from the needs of those who conceived spontaneously [32]. As there was no existing evidence available, recommendations were based on “good practice points” informed by expert opinion. In summary, fertility staff should refer or offer additional psychosocial care to patients at increased risk of experiencing psychosocial distress or problems, or to discuss their worries about the pregnancy [21].

German guidelines provided information for psychosocial counseling in the area of gamete donation, specific to donor insemination, as this is the only legal form of gamete donation in Germany [20]. There are several complexities which are associated with building a family with the assistance of donated semen which differ from building a family with gametes of both intended parents, including the differences between biological and social parenthood, how this affects the members of the intended family and any family of the donor, and how it impacts the future child. One recommendation is specific to how both partners may feel toward the donated semen and suggests that counselling can help towards understanding and managing these feelings.

## Discussion

Few existing CPGs for women pregnant following ART were identified. A total of 10 guidelines were included, with a focus on models of care, risks of ART, screening in pregnant women, care of women with conditions not specific to those pregnant using ART, and psychosocial counselling for those involved in ART. The associated degree of rigor based on formal quality assessments using the AGREE-II tool was found to be both variable and limited; only one CPG, the ESHRE guideline, referenced a full publication [32], which provided additional information on several key considerations (methodology, the process of external review, and information for updating) which led to a notably higher score. Efforts should be made to improve the quality of future guidelines since rigorously developed CPGs have shown to improve healthcare processes and patient outcomes [42].

Recommendations within these guidelines related to the population were often based on observational studies and expert opinion. Many of the recommendations were indicated to be the same as for the care suggested for women who conceive spontaneously. This was either due to a lack of evidence specific to women who conceived with ART, conflicting evidence, or because the evidence suggested that there was little or no difference between these women. Additionally, the recommendations from the ACCP CPG were based on some evidence on women who were undergoing ART treatment and not yet pregnant, however, this evidence was combined with studies that included women who were followed through pregnancy.

Several fundamental points of care can be drawn from the CPGs that were reviewed. One of the main differences of care for women becoming pregnant using ART (relative to those conceiving spontaneously) was for those who became pregnant with IVF-ICSI, because of a higher risk of birth defects [34, 35]. In these cases, prenatal diagnosis screening is recommended [43]. Of note, none of the guidelines discussed about Prenatal Genetic Screening/Prenatal Genetic Diagnosis (PGS/PGD) and perinatal outcomes, a more recent technology that needs to be addressed in future CPGs. In addition, there is sufficient evidence that multi-fetal pregnancies (triplets or more) contribute to high risk pregnancies, deliveries, and poorer outcomes [44, 45]. These pregnancies should be avoided, and if they occur, the option of selective reduction should be discussed, including the emotions associated with this and the possibility of loss of the entire pregnancy [46]. A common theme throughout many of the guidelines was the need for counselling prior to treatment, during treatment, and during pregnancy. Many of the decisions that are to be made in the course of ART treatment should be discussed with qualified health professionals, including the risks associated with treatment (e.g. multi-fetal pregnancies) [45], risks during pregnancy (e.g., preeclampsia) [47], higher risk deliveries (e.g., postpartum hemorrhage) [48], and risk to the baby (e.g., low birth weight, pre-term delivery) [49]. It should be noted that not all ART methods were addressed in the set of included CPGs (e.g., no specific mention for surrogate mothers). There may be a need to consider different care options depending on the different types of ART, as some techniques are associated with additional risks. For example, some evidence indicates that there is an increased risk of preeclampsia with IVF but not for IUI compared to spontaneous conception [47]. Others have reported a higher risk of preeclampsia in women conceiving by IUI with donor sperm compared with partner sperm [50]. Comparing pregnancies with donor oocytes to autologous IVF, there is an increased risk for pregnancy-induced hypertension [51] and preeclampsia [52].

Important gaps were noticed. Clear indications for directing women pregnant using ART to low- versus high-risk antenatal care do not exist. Furthermore, consensus regarding ideal antenatal care (including details such as types and timing of screening tests, medication and supplement dosing and timing of delivery) of these women is lacking in both low-risk and high-risk settings. Although it is recognized that there is a higher incidence of mono-chorionic twinning with IVF than the general population, none of the guidelines addressed the use of early ultrasound to determine twin chorionicity. In relation to evidence-base recommendations known to decrease adverse pregnancy outcomes in women at high risk, none of the identified CPGs considered folic acid supplementation to decrease the risk of congenital anomalies in ART pregnancies, or the use of low-dose aspirin to decrease the risk of preeclampsia. Moreover, recent guidelines specific to these two recommendations in the general obstetrical population, did not identify ART pregnancies as a high risk population who could benefit from an adjusted dose of folic acid [53], or low-dose aspirin after 12 weeks of gestation [54]. Finally, we did not identify guidelines addressing interventions during delivery (e.g. induction of labor versus spontaneous labor onset, cesarean section versus vaginal delivery) in pregnancies conceived through ART.

## Conclusion

There is evidence that supports an increased risk of adverse maternal and perinatal outcomes in pregnancies conceived using ART. The underlying cause of infertility may play a role, and even within ART, there appears to be different levels of risk depending on the technology used. As this quality of evidence grows and improves, CPGs specific to this population need to be re-evaluated. Although the quality of most included guidelines were deemed to require modifications (e.g., provide additional details of methodology), it is recommended that women who conceive using ART should be followed by health care providers with a recognized postgraduate qualification in obstetrics, be offered appropriate screening and diagnostic tests, and have access to psychosocial counselling throughout the entire process. The benefit of current recommendations known to decrease the risk of congenital malformations and preeclampsia in the general obstetrical population should be evaluated in ART pregnancies.

## Additional files

Additional file 1: Search Strategy for Pregnancy using ART – Guidelines/Care Plans (DOCX 18 kb)

Additional file 2: Excluded studies with reasons (DOCX 38 kb)

## Abbreviations

ACCP: American College of Chest Physicians
ACOG: American College of Obstetricians and Gynecologists
AFT: Alfa fetoprotein
AGREE: Appraisal of Guidelines for Research & Evaluation
ANC: Antenatal care
aOR: Adjusted Odds Ratio
ART: Assisted Reproductive Technologies
CPGs: Clinical Practice Guidelines
E3: Estradiol
ESHRE: European Society of Human Reproduction and Embryology
GIFT: Gamete intrafallopian transfer
GRADE: Grading of Recommendations, Assessment, Development, and Evaluation
HCG: Human chorionic gonadotropin
HR: Hazard Ratio
ICSI: Intracytoplasmic Sperm Injection
IUI: Intra-Uterine Insemination
IVF: In Vitro Fertilization
OHSS: Ovarian Hyperstimulation Syndrome
PAPP-A: Pregnancy associated plasma protein-A
PGD: Prenatal Genetic Diagnosis
PGS: Prenatal Genetic Screening
PRISMA: Preferred Reporting Items for Systematic Reviews and Meta-Analyses
SOGC: Society of Obstetricians and Gynaecologists of Canada
VTE: venous thromboembolism
