# Supplementary material for: Care plans for women pregnant using assisted reproductive technologies: a systematic review
Source: Reprod Health. 2019 Jan 29;16:9. doi: 10.1186/s12978-019-0667-z (PMC6352361; doi:10.1186/s12978-019-0667-z)
Supplement: Supplementary file 1 — Search Strategy for Pregnancy using ART – Guidelines/Care Plans (DOCX 18 kb) [file 12978_2019_667_MOESM1_ESM.docx]

# Appendix 1: Search Strategy for Pregnancy using ART – Guidelines/Care Plans

Database: Embase Classic+Embase <1947 to 2017 June 12>, Ovid MEDLINE(R) Epub Ahead of Print, In-Process & Other Non-Indexed Citations, Ovid MEDLINE(R) Daily and Ovid MEDLINE(R) <1946 to Present>

Search Strategy:

--------------------------------------------------------------------------------

1 Pregnancy/ (1476998)

2 exp Pregnancy Complications/ (521935)

3 Pregnant Women/ (55869)

4 exp Pregnancy Trimesters/ (750932)

5 pregnan*.tw,kw. (1066546)

6 Prenatal Care/ (57766)

7 (prenatal* or antenatal* or ante natal* or antepartum or ante partum).tw,kw. (276574)

8 or/1-7 [PREGNANCY, PREGNANT WOMEN] (1976062)

9 exp Reproductive Techniques, Assisted/ (147941)

10 (assist* adj3 (conceiv* or concepti* or reproducti*)).tw,kw. (34757)

11 (reproducti* adj3 (technic or technics or technique*)).tw,kw. (7584)

12 ART.tw,kw. (170274)

13 ((artificial* or intrauterine or intra-uterine) adj3 inseminat*).tw,kw. (19213)

14 IUI.tw,kw. (4299)

15 ("In Vitro" adj3 (fertili* or reproducti*)).tw,kw. (53874)

16 IVF.tw,kw. (55183)

17 "IVF-ET".tw,kw. (5106)

18 (test-tube* adj3 (baby or babies or fertili* or reproducti*)).tw,kw. (392)

19 (mitochondrial replacement adj3 (technic or technics or technique* or therap* or treat*)).tw,kw. (98)

20 ((pronuclear or pro-nuclear or spindle) adj3 (transfer technic* or transfer technique*)).tw,kw. (4)

21 (donor adj3 (conceiv* or conception*)).tw,kw. (703)

22 ((embryo or embryos or blastocyst*) adj5 (transfer* or transplant*)).tw,kw. (44583)

23 ((embryo or embryos or blastocyst*) adj5 (cryotransfer* or cryo-transfer* or cryotransplant* or cryo-transplant*)).tw,kw. (31)

24 ((embryo or embryos or blastocyst*) and (eSet? or dSet? or eDet? or eFet? or eMet?)).tw,kw. (526)

25 ("IVF-SET" or "IVF-DET" or "IVF-FET" or "IVF-MET" or "IVF-eSET" or "IVF-dSET" or "IVF-eDET" or "IVF-eFET" or "IVF-eMET").tw,kw. (58)

26 ("ICSI-SET" or "ICSI-DET" or "ICSI-FET" or "ICSI-MET" or "ICSI-eSET" or "ICSI-dSET" or "ICSI-eDET" or "ICSI-eFET" or "ICSI-eMET").tw,kw. (20)

27 ((embryo or embryos or blastocyst* or elective or non-elective) adj3 (SET? or SBT? or DET? or DBT? or FET? or FBT? or MET? or MBT? or 2BT?)).tw,kw. (2925)

28 (gamete adj (intrafallopian or intra-fallopian) adj transfer*).tw,kw. (1371)

29 ("in vitro" adj3 matur*).tw,kw. and (exp Oocytes/ or oocyte*.tw,kw.) (11562)

30 ((oocyte* or ova or ovum) adj3 (donat* or donor*)).tw,kw. (6821)

31 (zygote* adj (intrafallopian or intra-fallopian) adj transfer*).tw,kw. (249)

32 ((pronuclear or pro-nuclear) adj stage adj2 transfer*).tw,kw. (59)

33 PROST.tw,kw. (218)

34 ZIFT.tw,kw. (231)

35 (ovar* adj3 stimulat*).tw,kw. (18001)

36 (ovulat* adj3 (induc* or stimulat*)).tw,kw. (20190)

37 (sperm adj2 (inject* or microinject* or micro-inject*) adj3 (intracytoplasmic or intra-cyctoplasmic)).tw,kw. (14665)

38 ICSI.tw,kw. (20844)

39 Surrogate Mothers/ (1685)

40 ((surrogate or gestation* or host) adj2 (carrier* or mother*)).tw,kw. (4205)

41 (surrogate adj2 pregnan*).tw,kw. (157)

42 exp Infertility/th (26017)

43 ((infertil* or subfecund* or sub-fecund* or subfertil* or sub-fertil*) adj5 (manag* or therap* or treat*)).tw,kw. (26987)

44 or/9-43 [ASSISTED REPRODUCTION] (396151)

45 8 and 44 [ART & PREGNANCY] (113745)

46 exp Animals/ not (exp Animals/ and Humans/) (15300560)

47 45 not 46 [ANIMAL-ONLY REMOVED] (75559)

48 (comment or editorial or interview or news or newspaper article).pt. (1753470)

49 (letter not (letter and randomized controlled trial)).pt. (1947757)

50 47 not (48 or 49) [OPINION PIECES REMOVED] (72296)

51 exp Guidelines as Topic/ (552160)

52 exp Clinical Protocols/ (235459)

53 Guideline.pt. (16560)

54 Practice Guideline.pt. (23350)

55 standards.fs. (643196)

56 Consensus Development Conference.pt. (10660)

57 Consensus Development Conference, NIH.pt. (779)

58 (guidance* or guideline* or standards or recommendation*).ti. (288269)

59 (expert consensus or consensus statement* or consensus conference* or practice parameter* or position statement* or policy statement* or CPG or CPGs).tw,kw. (102524)

60 ((care or clinical or healthcare or patient or practice or therap* or treatment*) adj2 (algorithm* or protocol*)).tw,kw. (105553)

61 Critical Pathways/ (13223)

62 pathway*.tw,kw. (2011454)

63 ((care or clinical or critical or healthcare) adj2 (map or maps or path or paths)).tw,kw. (3966)

64 (care plan? or healthcare plan?).tw,kw. (17310)

65 patient care/ (254334)

66 exp "Continuity of Patient Care"/ (870972)

67 ((continuit* or continuum) adj3 (care or healthcare)).tw,kw. (23183)

68 or/51-67 [GUIDELINES, CARE PLANS] (4402457)

69 50 and 68 [ART IN PREGNANCY GUIDELINES/CARE PLANS] (4823)

70 limit 69 to yr="2007-current" (2344)

71 70 use ppez [MEDLINE RECORDS] (1299)

72 pregnancy/ (1476998)

73 exp pregnancy complication/ (521935)

74 pregnant woman/ (68517)

75 first trimester pregnancy/ or second trimester pregnancy/ or third trimester pregnancy/ (100151)

76 pregnan*.tw,kw. (1066546)

77 prenatal care/ (57766)

78 (prenatal* or antenatal* or ante natal* or antepartum or ante partum).tw,kw. (276574)

79 or/72-78 [PREGNANCY, PREGNANT WOMEN] (1970044)

80 exp infertility therapy/ (86070)

81 (assist* adj3 (conceiv* or concepti* or reproducti*)).tw,kw. (34757)

82 (reproducti* adj3 (technic or technics or technique*)).tw,kw. (7584)

83 ART.tw,kw. (170274)

84 ((artificial* or intrauterine or intra-uterine) adj3 inseminat*).tw,kw. (19213)

85 IUI.tw,kw. (4299)

86 ("In Vitro" adj3 (fertili* or reproducti*)).tw,kw. (53874)

87 IVF.tw,kw. (55183)

88 "IVF-ET".tw,kw. (5106)

89 (test-tube* adj3 (baby or babies or fertili* or reproducti*)).tw,kw. (392)

90 (mitochondrial replacement adj3 (technic or technics or technique* or therap* or treat*)).tw,kw. (98)

91 ((pronuclear or pro-nuclear or spindle) adj3 (transfer technic* or transfer technique*)).tw,kw. (4)

92 (donor adj3 (conceiv* or conception*)).tw,kw. (703)

93 ((embryo or embryos or blastocyst*) adj5 (transfer* or transplant*)).tw,kw. (44583)

94 ((embryo or embryos or blastocyst*) adj5 (cryotransfer* or cryo-transfer* or cryotransplant* or cryo-transplant*)).tw,kw. (31)

95 ((embryo or embryos or blastocyst*) and (eSet? or dSet? or eDet? or eFet? or eMet?)).tw,kw. (526)

96 ("IVF-SET" or "IVF-DET" or "IVF-FET" or "IVF-MET" or "IVF-eSET" or "IVF-dSET" or "IVF-eDET" or "IVF-eFET" or "IVF-eMET").tw,kw. (58)

97 ("ICSI-SET" or "ICSI-DET" or "ICSI-FET" or "ICSI-MET" or "ICSI-eSET" or "ICSI-dSET" or "ICSI-eDET" or "ICSI-eFET" or "ICSI-eMET").tw,kw. (20)

98 ((embryo or embryos or blastocyst* or elective or non-elective) adj3 (SET? or SBT? or DET? or DBT? or FET? or FBT? or MET? or MBT? or 2BT?)).tw,kw. (2925)

99 (gamete adj (intrafallopian or intra-fallopian) adj transfer*).tw,kw. (1371)

100 ("in vitro" adj3 matur*).tw,kw. and (exp Oocytes/ or oocyte*.tw,kw.) (11562)

101 ((oocyte* or ova or ovum) adj3 (donat* or donor*)).tw,kw. (6821)

102 (zygote* adj (intrafallopian or intra-fallopian) adj transfer*).tw,kw. (249)

103 ((pronuclear or pro-nuclear) adj stage adj2 transfer*).tw,kw. (59)

104 PROST.tw,kw. (218)

105 ZIFT.tw,kw. (231)

106 (ovar* adj3 stimulat*).tw,kw. (18001)

107 (ovulat* adj3 (induc* or stimulat*)).tw,kw. (20190)

108 (sperm adj2 (inject* or microinject* or micro-inject*) adj3 (intracytoplasmic or intra-cyctoplasmic)).tw,kw. (14665)

109 ICSI.tw,kw. (20844)

110 ((surrogate or gestation* or host) adj2 (carrier* or mother*)).tw,kw. (4205)

111 (surrogate adj2 pregnan*).tw,kw. (157)

112 ((infertil* or subfecund* or sub-fecund* or subfertil* or sub-fertil*) adj5 (manag* or therap* or treat*)).tw,kw. (26987)

113 or/80-112 [ASSISTED REPRODUCTION] (373616)

114 79 and 113 [ART & PREGNANCY] (104629)

115 exp animal experimentation/ or exp models animal/ or exp animal experiment/ or nonhuman/ or exp vertebrate/ (45547365)

116 exp human/ or exp human experimentation/ or exp human experiment/ (35639533)

117 115 not 116 (9909534)

118 114 not 117 [ANIMAL-ONLY REMOVED] (90883)

119 editorial.pt. (979387)

120 letter.pt. not (letter.pt. and randomized controlled trial/) (1943063)

121 118 not (119 or 120) [OPINION PIECES REMOVED] (88763)

122 exp practice guideline/ (434591)

123 (guidance* or guideline* or standards or recommendation*).ti. (288269)

124 (expert consensus or consensus statement* or consensus conference* or practice parameter* or position statement* or policy statement* or CPG or CPGs).tw,kw. (102524)

125 ((care or clinical or healthcare or patient or practice or therap* or treatment*) adj2 (algorithm* or protocol*)).tw,kw. (105553)

126 pathway*.tw,kw. (2011454)

127 ((care or clinical or critical or healthcare) adj2 (map or maps or path or paths)).tw,kw. (3966)

128 (care plan? or healthcare plan?).tw,kw. (17310)

129 patient care/ (254334)

130 ((continuit* or continuum) adj3 (care or healthcare)).tw,kw. (23183)

131 or/122-130 [GUIDELINES/CARE PATHWAYS] (3056629)

132 121 and 131 [ART IN PREGNANCY - GUIDELINES/CARE PATHWAYS] (4009)

133 conference abstract.pt. (2568950)

134 132 not 133 [CONFERENCE ABSTRACTS REMOVED] (3340)

135 limit 134 to yr="2007-current" (2062)

136 135 use emczd [EMBASE RECORDS] (1460)

137 71 or 136 [BOTH DATABASES] (2759)

138 remove duplicates from 137 (2173) [TOTAL UNIQUE RECORDS]

139 138 use ppez (1228) [MEDLINE UNIQUE RECORDS]

140 from 139 keep 1-1000 (1000)

141 from 139 keep 1001-1228 (228)

142 138 use emczd (945) [EMBASE UNIQUE RECORDS]

***************************
