# Supplementary material for: Care plans for women pregnant using assisted reproductive technologies: a systematic review
Source: Reprod Health. 2019 Jan 29;16:9. doi: 10.1186/s12978-019-0667-z (PMC6352361; doi:10.1186/s12978-019-0667-z)
Supplement: Supplementary file 2 — Excluded studies with reasons (DOCX 38 kb) [file 12978_2019_667_MOESM2_ESM.docx]

# Appendix 2: Excluded studies with reasons

## Full text not available (n=8)

1. Nuzback K. A lost art. *Texas Medicine* 2014; 110(6):41-44.
2. Tiras B, Cenksoy PO. Practice of embryo transfer: recommendations during and after. *Seminars in Reproductive Medicine* 2014; 32(4):291-296.
3. Belaisch-Allart J. [Assisted reproductive technologies and ethics]. *La Revue du praticien* 2014; 64(1):86-87.
4. Tur-Kaspa I. Clinical management of in vitro fertilization with preimplantation genetic diagnosis. *Seminars in Reproductive Medicine* 2012; 30(4):309-322.
5. Baston H. Use of technology in childbirth. 3. Assisted conception. *The Practising Midwife* 2011; 14(11):35-39.
6. 7th RQRUM Annual Meeting - Abstracts. *J Pop Therapeutics and Clin Pharma* 2011; 18(2):e275-e314.
7. Aura-Masip M, Rodriguez-Galvez I, Cabello Y, Carrera-Roig M, Del Rio BF, Mercader A, Fernandez M. Embryo tranfsfer and luteal phase support: The final step in in vitro fertilisation treatment. *Medicina Reproductiva Embriologia Clinica* 2016; 1(3):24-36.

RANZCOG 2016 Annual Scientific Meeting. *Australian and New Zealand Journal of Obstetrics and Gynaecology* 2016; 56.

## Article not in English or French (n=11)

1. Kimura K, Iwamoto M, Tanaka S, Watanabe T, Aihara T, Sugimoto T, *et al*. [Is the LHRH Agonist Recommended for Fertility Preservation ?] Gan to kagaku ryoho. *Cancer & Chemotherapy* 2015; 42(8):1019-1021.
2. Lindqvist PG, Rova K, Thurn L, Wegnelius G, Nord E, Hellgren M. [Venous thrombosis in pregnancy and assisted reproduction. Updated recommendations on risk assessment and indications for thromboprophylaxis]. *Lakartidningen* 2014; 111(32-33):1305-1308.
3. Wennberg AL, Lundborg E, Zachrisson U, Nielsen S, Saldeen P, Brannstrom M. [Private egg bank to protect fertility against aging]. *Lakartidningen* 2014; 111(18-19):793-795.
4. Polish Gynecological Society [Statement of the Polish Gynecological Society on the application of myo-inozytol in patients with PCOS (polycystic ovary syndrome)]. *Ginekologia polska* 2014; 85(2):158-160.
5. Zespol Ekspertow Polskiego Towarzystwa Ginekologicznego [Statement of the Polish Gynecological Society Expert Group on the prevention of iron deficiency and of anemia caused by iron deficiency with a low dose heme iron in women. State of the art, 2013]. *Ginekologia polska* 2014; 85(1):74-78.
6. Zakova J, Travnik P, Malenovska A, Huttelova R. [Activities and responsibilities of workers in embryologic and andrologic laboratories in assisted reproduction centers]. *Ceska gynekologie* 2013; 78(5):481-484.
7. Hubalewska-Dydejczyk A, Lewinski A, Milewicz A, Radowicki S, Poreba R, Karbownik-Lewinska M, Kostecka-Matyja M, Trofimiuk-Muldner M, Pach D, Zygmunt A, Bandurska-Stankiewicz E, Bar-Andziak E, Bednarczuk T, Buziak-Bereza M, Drews K, Gietka-Czernel M, Gorska M, Jastrzebska H, Junik R, Nauman J, Niedziela M, Reron A, Sworczak K, Syrenicz A, Zgliczynski W. [Management of thyroid diseases during pregnancy]. *Endokrynologia Polska* 2011; 62(4):362-381.
8. Kably Ambe A, Lopez Ortiz CS, Serviere Zaragoza C, Velazquez Cornejo G, Perez Pena E, Santos Haliscack R, Luna Rojas M, Valerio E, Santana H, Gavino Gavino F. [Mexican National Consensus on Assisted Reproduction Treatment]. *Ginecologia y obstetricia de Mexico* 2012; 80(9):581-624.
9. Gjerris AC, Loft A, Pinborg AB, Christiansen M, Tabor A. [Prenatal screening and diagnostics after infertility treatment]. *Ugeskrift for laeger* 2007; 169(5):414-419.
10. Soderstrom-Anttila V. [Surrogate motherhood--experienced team should follow the entire process]. Lakartidningen 2014; 111(18-19):802-804.
11. Radakovic B. New strategies and recommendation European community in treatment of infertility with assisted reproductive technologies. *Gynaecologia et Perinatologia* 2009; 18(2):55-60.

## Does not discuss care given to women during pregnancy or delivery (n=86)

1. Farquhar C, Marjoribanks J, Brown J, Fauser BCJM, Lethaby A, Mourad S, Rebar R, Showell M, van der Poel S. Management of ovarian stimulation for IVF: narrative review of evidence provided for World Health Organization guidance. *Reproductive Biomedicine Online* 2017; 35:3-16.
2. Messini CI, Daponte A, Anifandis G, Mahmood T, Messinis IE. Standards of Care in infertility in Europe. *European Journal of Obstetrics, Gynecology, and Reproductive Biology* 2016; 207:205-210.
3. Rienzi L, Gracia C, Maggiulli R, LaBarbera AR, Kaser DJ, Ubaldi FM, Vanderpoel S, Racowsky C. Oocyte, embryo and blastocyst cryopreservation in ART: systematic review and meta-analysis comparing slow-freezing versus vitrification to produce evidence for the development of global guidance. *Human Reproduction Update* 2017; 23(2):139-155.
4. Ahemmed B, Sundarapandian V, Gutgutia R, Balasubramanyam S, Jagtap R, Biliangady R, Gupta P, Jadhav S, Satwik R, Dewda PR, Thakor P, Esteves SC Outcomes and Recommendations of an Indian Expert Panel for Improved Practice in Controlled Ovarian Stimulation for Assisted Reproductive Technology. *International Journal of Reproductive Medicine* 2017; Article ID 9451235.
5. Jindal Sangita K, Rawlins RG, Muller CH, Drobnis EZ. Guidelines for risk reduction when handling gametes from infectious patients seeking assisted reproductive technologies. *Reproductive Biomedicine Online* 2016; 33(2):121-130.
6. Balen AH, Morley LC, Misso M, Franks S, Legro RS, Wijeyaratne CN, Stener-Victorin E, Fauser BCJM, Norman RJ, Teede H. The management of anovulatory infertility in women with polycystic ovary syndrome: an analysis of the evidence to support the development of global WHO guidance. *Human Reproduction Update* 2016; 22(6):687-708.
7. MacArthur T, Bachmann G, Ayers C. Menopausal women requesting egg/embryo donation: examining health screening guidelines for assisted reproductive technology. *Menopause* (New York, N.Y.) 2016; 23(7):799-802.
8. Lee MS, Evans BT, Stern AD, Hornstein MD. Economic implications of the Society for Assisted Reproductive Technology embryo transfer guidelines: healthcare dollars saved by reducing iatrogenic triplets. *Fertility and Sterility* 2016; 106(1):189-195.
9. Liu RM, Liu HJ, Cong JL, Sun AL, Du JD, Sun CM. Genetic characteristics of the couple with non-syndromic sensorineural hearing loss and fertility guidance. *International Journal of Clinical and Experimental Medicine* 2015; 8(11):21746-21754.
10. Dokuzeylul N. Antagonist use in intrauterine insemination (IUI) cycles. *Journal of the Turkish German Gynecological Association* 2009; 10(4):226-231.
11. Practice Committee of the American Society for Reproductive Medicine and Practice Committee of the American Society for Reproductive Medicine Performing the embryo transfer: a guideline. *Fertility and Sterility* 2017; 107(4):882-896.
12. Practice Committee of the American Society for Reproductive Medicine and Practice Committee of the Society for Assisted Reproductive Technology Guidance on the limits to the number of embryos to transfer: a committee opinion. *Fertility and Sterility* 2017; 107(4):901-903.
13. Practice Committee of the American Society for Reproductive Medicine. Penzias A, Bendikson K, Butts S, Coutifaris C, Falcone T, Fossum G, Gitlin S, Gracia C, Hansen K, Mersereau J, Odem R, Rebar R, Reindollar R, Rosen M, Sandlow J, Vernon M. ASRM standard embryo transfer protocol template: a committee opinion. *Fertility and Sterility* 2017; 107(4):897-900.
14. Toner JP, Coddington CC, Doody K, Van Voorhis B, Seifer DB, Ball GD, Luke B, Wantman E. Society for Assisted Reproductive Technology and assisted reproductive technology in the United States: a 2016 update. *Fertility and Sterility* 2016; 106(3):541-546.
15. Practice Committee of the American Society for Reproductive Medicine and Practice Committee of the American Society for Reproductive Medicine Uterine septum: a guideline. *Fertility and Sterility* 2016; 106(3):530-540.
16. Di Martino D, Cetin I, Frusca T, Ferrazzi E, Fuse' F, Gervasi MT, Plebani M, Todros T. Italian Advisory Board: sFlt-1/PlGF ratio and preeclampsia, state of the art and developments in diagnostic, therapeutic and clinical management. *European Journal of Obstetrics, Gynecology, and Reproductive Biology* 2016; 206:70-73.
17. Oktay K, Bedoschi G, Berkowitz K, Bronson R, Kashani B, McGovern P, Pal L, Quinn G, Rubin K. Fertility Preservation in Women with Turner Syndrome: A Comprehensive Review and Practical Guidelines. *Journal of Pediatric and Adolescent Gynecology* 2016; 29(5):409-416.
18. Ebina Y, Katabuchi H, Mikami M, Nagase S, Yaegashi N, Udagawa Y, Kato H, Kubushiro K, Takamatsu K, Ino K, Yoshikawa H. Japan Society of Gynecologic Oncology guidelines 2013 for the treatment of uterine body neoplasms. *International Journal of Clinical Oncology* 2016; 21(3):419-434.
19. Nau JY. [Not Available]. *Revue medicale suisse* 2016; 12(516):862-863.
20. Fragouli, Elpida First validated clinical test selects best embryos for IVF and viable pregnancies. *Medical Laboratory Observer* 2016; 48(2): 38-.
21. Cedars MI. Assisted reproductive technology: moving forward--or just moving? *Fertility and Sterility* 2016; 105(3):588-589.
22. Janicka A, Spaczyniski RZ, Kurzawa R, SPiN PTG, Fertility, Clinics, and Polish Gynaecological Society Assisted reproductive medicine in Poland --Fertility and Sterility Special Interest Group of the Polish Gynaecological Society (SPiN PTG) 2012 report. *Ginekologia polska* 2015; 86(12):932-939.
23. Goodman NF, Cobin RH, Futterweit W, Glueck JS, Legro RS, Carmina E, American Association of Clinical Endocrinologists (AACE), American College of Endocrinology (ACE), and Androgen Excess and PCOS Society. Disease state clinical review: Guide to the best practices in the evaluation and treatment of polycystic ovary syndrome – Part 2. *Endocrine Practice* 2015; 21(12):1415-1426.
24. Harbottle S, Hughes C, Cutting R, Roberts S, Brison D, and Association Of Clinical Embryologists & The (ACE) British Fertility Society (BFS) Elective Single Embryo Transfer: an update to UK Best Practice Guidelines. *Human Fertility* (Cambridge, England) 2015; 18(3):165-183.
25. Rodolakis, Alexandros, Biliatis, Ioannis, Morice, Philippe, Reed, Nick, Mangler, Mandy, Kesic, Vesna, and Denschlag, Dominik European Society of Gynecological Oncology Task Force for Fertility Preservation: Clinical Recommendations for Fertility-Sparing Management in Young Endometrial Cancer Patients. *International Journal of Gynecological Cancer* 2015; 25(7):1258-1265.
26. Borowski KS, Brost BC, Stewart EA, Hay EJ, Coddington CC. Preconception risk stratification before fertility care. *Fertility and Sterility* 2015; 104(1):28-31.
27. Rice S. Inside the gray zone. New treatments of questionable effectiveness pose cost, safety questions. *Modern Healthcare* 2015; 45(4):12-14.
28. Nardo LG, El-Toukhy T, Stewart J, Balen AH, Potdar N. British Fertility Society Policy and Practice Committee: adjuvants in IVF: evidence for good clinical practice. *Human Fertility* (Cambridge, England) 2015; 18(1):2-15.
29. Practice Committee of the American Society for Reproductive Medicine and Practice Committee of the Society for Assisted Reproductive Technology Recommendations for practices utilizing gestational carriers: a committee opinion. *Fertility and Sterility* 2015; 103(1):e1-e8.
30. Grifo J, Kofinas J, Schoolcraft WB. The practice of in vitro fertilization according to the published literature. *Fertility and Sterility* 2014; 102(3):658-659.
31. Practice Committee of the American Society for Reproductive Medicine and Practice Committee of the Society for Assisted Reproductive Technology Role of assisted hatching in in vitro fertilization: a guideline. *Fertility and Sterility* 2014; 102(2):348-351.
32. Johnston J, Gusmano MK, Patrizio P. Preterm births, multiples, and fertility treatment: recommendations for changes to policy and clinical practices. *Fertility and Sterility* 2014; 102(1):36-39.
33. O'Flynn N. Assessment and treatment for people with fertility problems: NICE guideline. *The British Journal of General Practice* 2014; 64(618):50-51.
34. den Breejen EME, Nelen WLDM, Schol SFE, Kremer JAM, Hermens RPMG. Development of guideline-based indicators for patient-centredness in fertility care: what patients add. *Human Reproduction* (Oxford, England) 2013; 28(4):987-996.
35. Haagen EC, Nelen WLDM, Adang EM, Grol RPTM, Hermens RPMG, Kremer JAM. Guideline adherence is worth the effort: a cost-effectiveness analysis in intrauterine insemination care. *Human Reproduction* (Oxford, England) 2013; 28(2):357-366.
36. FIGO Committee for Ethical Aspects of Human Reproduction and Women's Health FIGO Committee Report: Surrogacy. *International Journal of Gynaecology and Obstetrics* 2008; 102(3):312-313.
37. Vause TDR, Cheung AP, Sierra S, Claman P, Graham J, Guillemin JA, Lapensee L, Steward S, Wong BC-M, Society of Obstetricians and Gynaecologists of Canada Ovulation induction in polycystic ovary syndrome: No. 242, May 2010. *International Journal of Gynaecology and Obstetrics* 2010; 111(1):95-100.
38. Practice Committee of the American Society for Reproductive Medicine and Practice Committee for the Society for Assisted Reproductive Technology Recommendations for practices utilizing gestational carriers: an ASRM Practice Committee guideline. *Fertility and Sterility* 2012; 97 (6): 1301-1308.
39. Katelaris A. How far have we come in 30 years of IVF? *The Medical Journal of Australia* 2011; 195(10):563-.
40. CNGOF. [Update of myoma management: guidelines for clinical practice - text of the guidelines]. *Journal de gynecologie, obstetrique et biologie de la reproduction* 2011; 40(8):953-961.
41. Adashi EY, Wyden R. Public reporting of clinical outcomes of assisted reproductive technology programs: implications for other medical and surgical procedures. *JAMA* 2011; 306(10):1135-1136.
42. Orentlicher D. Multiple embryo transfers: time for policy. *The Hastings Center report* 2010; 40(3):12-13.
43. Min JK. Hughes E, Young D, Gysler M, Hemmings R, Cheung AP, Goodrow GJ, Senikas V, Wong BC-M, Sierra S, Carranza-Mamane B, Case A, Dwyer C, Graham J, Havelock J, Lee F, Liu K, Vause T, Joint Society of Obstetricians and Gynaecologists of Canada-Canadian Fertility and Andrology Society Clinical Practice Guidelines Committee Elective single embryo transfer following in vitro fertilization. *Journal of Obstetrics and Gynaecology Canada* 2010; 32(4):363-377.
44. Harper J, Coonen E, De Rycke M, Fiorentino F, Geraedts J, Goossens V, Harton G, Moutou C, Pehlivan Budak T, Renwick P, Sengupta S, Traeger-Synodinos J, Vesela K. What next for preimplantation genetic screening (PGS)? A position statement from the ESHRE PGD Consortium Steering Committee. *Human Reproduction* (Oxford, England) 2010; 25(4):821-823.
45. Practice Committee of the American Society for Reproductive Medicine and Practice Committee of the Society for Assisted Reproductive Technology Guidelines on number of embryos transferred. *Fertility and Sterility* 2009; 92(5):1518-1519.
46. Association of Biomedical Andrologists, Association of Clinical Embryologists, British Andrology Society, British Fertility Society, and Royal College of Obstetricians and Gynaecologists UK guidelines for the medical and laboratory screening of sperm, egg and embryo donors (2008). *Human Fertility* (Cambridge, England) 2008; 11(4):201-210.
47. Germond M, Wirthner D, Senn A, Lausanne Consensus Meeting on National ART Registers Core data for assisted reproductive technology registers: results of a consensus meeting. *Reproductive Biomedicine Online* 2008; 17(6):834-840.
48. Fagot-Largeault A. [Human fertilisation and embryology authority]. *Medecine sciences* 2008; 24(11):997-1001.
49. Practice Committee of American Society for Reproductive Medicine and Practice Committee of Society for Assisted Reproductive Technology 2008 Guidelines for gamete and embryo donation: a Practice Committee report. *Fertility and Sterility* 2008; 90(5 Suppl):S30-S44.
50. Practice Committee of American Society for Reproductive Medicine and Practice Committee of Society for Assisted Reproductive Technology Blastocyst culture and transfer in clinical-assisted reproduction. *Fertility and Sterility* 2008; 90(5 Suppl):S174-S177.
51. Anderson RA, Pickering S. The current status of preimplantation genetic screening: British Fertility Society Policy and Practice Guidelines. *Human Fertility* (Cambridge, England) 2008; 11(2):71-75.
52. Cohen CB, Brandhorst B, Nagy A, Leader A, Dickens B, Isasi RM, Evans D, Knoppers BM. The use of fresh embryos in stem cell research: ethical and policy issues. *Cell Stem Cell* 2008; 2(5):416-421.
53. van Empel IWH, Nelen WLDM, Hermens RPMG, Kremer JAM. Coming soon to your clinic: high-quality ART. *Human Reproduction* (Oxford, England) 2008; 23(6):1242-1245.
54. Belaisch-Allart J. [Concerning the recommendations for good practice by the Afssaps. Medicines that induce ovulation: gonadotrophins (update, April 2007)]. *Gynecologie, obstetrique & fertilite* 2007; 35(9):917-922.
55. Mourad SM, Hermens RPMG, Nelen WLDM, Braat DDM, Grol RPTM, Kremer JAM. Guideline-based development of quality indicators for subfertility care. *Human Reproduction* (Oxford, England) 2007; 22(10):2665-2672.
56. Hamilton M. Multiple births in the United Kingdom--a consensus statement. *Human Fertility* (Cambridge, England) 2007; 10(2):71-74.
57. Jones HWJ, Cohen J. IFFS surveillance 07. *Fertility and Sterility* 2007; 87(4 Suppl 1):S1-67.
58. Johnson B, Chavkin W. Policy efforts to prevent ART-related preterm birth. *Maternal and Child Health Journal* 2007; 11(3):219-225.
59. Ziegler C. Excerpts from the World Medical Literature. *Journal of Obstetrics and Gynaecology Canada* 2017; 39(6):416-417.
60. Bahadur G, Homburg R, Al-Habib A. A New Dawn for Intrauterine Insemination: Efficient and Prudent Practice will Benefit Patients, the Fertility Industry and the Healthcare Bodies. *Journal of Obstetrics and Gynecology of India* 2017; 67(2):79-85.
61. Kwak-Kim J, Song J, Kim MWI, Gilman-Sachs A. Zika virus infection and biological treatment for reproductive medicine. *American Journal of Reproductive Immunology* 2017; 77(2):e12606-.
62. Malhotra A. Surrogacy in India at crossroads. *Journal of Reproductive Health and Medicine* 2016; 2:S16-S17.
63. Johnson, J. V. Obesity and infertility: The importance of ART policies and practice standards. *Fertility and Sterility* 2016; 105(3):602.
64. Grandone E, Villani M. Assisted reproductive technologies and thrombosis. *Thrombosis Research* 2015; 135(S1):S44-S45.
65. Coddington CC, Jensen JR. Multiple pregnancy: Changing expectations for patients and patterns for physicians. *Fertility and Sterility* 2015; 103(4):898-899.
66. Moragianni VA. Why are we still, 20 years later, depriving human immunodeficiency virus-serodiscordant couples of equal access to fertility care? *Fertility and Sterility* 2014; 102(2):352-353.
67. Coughlan C, Ola B. The subfertile couple. *Obstetrics, Gynaecology and Reproductive Medicine* 2013; 23(5):154-159.
68. Ory SJ. The national epidemic of multiple pregnancy and the contribution of assisted reproductive technology. *Fertility and Sterility* 2013; 100(4):929-930.
69. Shmorgun D, Claman P, Gysler M, Hemmings R, Cheung AP, Goodrow GJ, Hughes EG, Min JK, Roberts J, Senikas V, Wong BCM, Young DC. The diagnosis and management of ovarian hyperstimulation syndrome: No. 268, November 2011. *International Journal of Gynecology and Obstetrics* 2012; 116(3):268-773.
70. Hull J. The effects of acupuncture protocol modification on pregnancy rates when used as an adjunct to IVF. *Journal of Chinese Medicine* 2012; (99):47-54.
71. Raziel A, Schachter M, Friedler S, Ron-El R. Symposium: Update on prediction and management of OHSS - Outcome of IVF pregnancies following severe OHSS. *Reproductive Biomedicine Online* 2009; 19(1):61-65.
72. Bellver J, Pellicer A. Ovarian stimulation for ovulation induction and in vitro fertilization in patients with systemic lupus erythematosus and antiphospholipid syndrome. *Fertility and Sterility* 2009; 92(6):1803-1810.
73. Gleicher N, Oktay K, Barad DH. Patients are entitled to maximal IVF pregnancy rates. *Reproductive Biomedicine Onlin*e 2009; 18(5):599-602.
74. Zegers-Hochschild F, Adamson GD, de Mouzon J, Ishihara O, Mansour R, Nygren K, Sullivan E, Vanderpoel S. International Committee for Monitoring Assisted Reproductive Technology (ICMART) and the World Health Organization (WHO) revised glossary of ART terminology, 2009*. *Fertility and Sterility* 2009; 92(5):1520-1524.
75. Consensus on infertility treatment related to polycystic ovary syndrome. *Fertility and Sterility* 2008; 89 (3): 505-522.
76. Munne, S. Improving pregnancy outcome for IVF patients with preimplantation genetic screening. *Expert Review of Obstetrics and Gynecology* 2008; 3(5):635-646.
77. Gurgan, T. and Demirol, A. Unresolved issues regarding assisted reproduction technology. *Reproductive Biomedicine Online* 2007; 14(SUPPL. 1):40-43.
78. Rosenthal, L. and Anderson, B. Acupuncture and in vitro fertilisation: Recent research and clinical guidelines. *Journal of Chinese Medicine* 2007; (84):28-35.
79. Grey literature: Fertility Overview [NICE pathway]. NICE, 2017. <https://pathways.nice.org.uk/pathways/fertility>
80. Grey literature: Ovarian Hyperstimulation Syndrome, Management (Green-top Guideline No. 5). RCOG, 2016. <https://www.rcog.org.uk/en/guidelines-research-services/guidelines/gtg5/>
81. Grey literature: Hysteroscopic metroplasty of a uterine septum for primary infertility [Interventional Guidance 509]. NICE, Jan 2015. <https://www.nice.org.uk/guidance/ipg509>
82. Grey literature: Fertility Problems [Quality Standard 73]. NICE, 2014. <https://www.nice.org.uk/guidance/qs73>
83. Grey literature: The Prevention of Ovarian Hyperstimulation Syndrome. SOGC, 2014. <http://www.jogc.com/article/S1701-2163(15)30417-5/abstract?showall=true>
84. Grey literature: ACR Appropriateness Criteria. <https://www.guideline.gov/summaries/summary/49087/acr-appropriateness-criteria--infertility>
85. Grey literature: Fertility problems: assessment and treatment. NICE, 2013 Feb (updated 2016 Aug) [Clinical guideline no. 156]. <https://www.nice.org.uk/guidance/cg156>
86. Grey literature: Sexual and Gender Minority Peoples’ Recommendation for Assisted Human Reproduction Services. *J Obstet Gynecol Canada* 2014 Feb; 36(2):145-153.

## Women did not conceive with ART (n=15)

1. Martinez Lopez JA, Garcia Vivar ML, Caliz R, Freire M, Galindo M, Hernandez MV, *et al.* Recommendations for the evaluation and management of patients with rheumatic autoimmune and inflammatory diseases during the reproductive age, pregnancy, postpartum and breastfeeding. *Reumatologia clinica* 2017 (article in press).
2. Andreoli L, Bertsias GK, Agmon-Levin N, Brown S, Cervera R, Costedoat-Chalumeau N, *et al*. EULAR recommendations for women's health and the management of family planning, assisted reproduction, pregnancy and menopause in patients with systemic lupus erythematosus and/or antiphospholipid syndrome. *Annals of the Rheumatic Diseases* 2017; 76(3):476-485.
3. Stepan H, Kuse-Fohl S, Klockenbusch W, Rath W, Schauf B, Walther T, Schlembach D. Diagnosis and Treatment of Hypertensive Pregnancy Disorders. Guideline of DGGG (S1-Level, AWMF Registry No.015/018, December 2013). *Geburtshilfe und Frauenheilkunde* 2015; 75(9):900-914.
4. Vagelli, Roberta, Tani, Chiara, and Mosca, Marta Pregnancy and menopause in patients with systemic lupus erythematosus and/or antiphospholipid syndrome. Practical messages from the EULAR guidelines. *Polish Archives of Internal Medicine* 2017; 127(2):115-121.
5. Practice Committee of the American Society for Reproductive Medicine Subclinical hypothyroidism in the infertile female population: a guideline. *Fertility and Sterility* 2015; 104(3):545-553.
6. Mintziori G, Lambrinoudaki I, Kolibianakis EM, Ceausu I, Depypere H, Erel C, *et al.* EMAS position statement: Late parenthood. *Maturitas* 2013; 76(2):200-204.
7. Liu K, Case A, Cheung AP, Sierra S, AlAsiri S, Carranza-Mamane B, Dwyer C, Graham J, Havelock J, Hemmings R, Lee F, Murdock W, Senikas V, Vause TDR, Wong BC-M. Advanced reproductive age and fertility: no. 269, November 2011. *International Journal of Gynaecology and Obstetrics* 2012; 117(1):95-102.
8. Loutfy MR, Margolese S, Money DM, Gysler M, Hamilton S, Yudin MH, Society of Obstetricians and Gynaecologists of Canada. Canadian HIV pregnancy planning guidelines. *JOGC* 2012; 34 (6):575-590.
9. Pentheroudakis G, Orecchia R, Hoekstra HJ, Pavlidis N, ESMO Guidelines Working Group Cancer, fertility and pregnancy. ESMO Clinical Practice Guidelines for diagnosis, treatment and follow-up. *Annals of Oncology* 2010; 21(Suppl 5):v266-v273.
10. Conard J, Horellou MH, Samama MM, American College of Chest Physicians (ACCP). [Pregnancy and venous thromboembolism. North-American and European guidelines. American College of Chest Physicians]. *Journal des maladies vasculaires* 2009; 34(5):300-313.
11. Thorogood M, Seed M, De Mott K, Guideline Development Group Management of fertility in women with familial hypercholesterolaemia: summary of NICE guidance. *BJOG* 2009; 116(4):478-479.
12. Practice Committee of American Society for Reproductive Medicine Guidelines for reducing the risk of viral transmission during fertility treatment. Fertility and sterility 2008; 90 (5 Suppl): S156-S162.
13. Redford C, Brooke A. Endocrinology update. *Clinical Medicine, Journal of the Royal College of Physicians of London* 2017; 17(1):37-39.
14. Deruelle P, Coulon C, Vaast P, Houfflin-Debarge V. Twin pregnancies: Where should we manage them? Where should they deliver? *Journal de Gynecologie Obstetrique et Biologie de la Reproduction* 2009; 38(8 SUPPL. 1):S56-S60.
15. Raine-Fenning N, Hopkisson J. Management of ectopic pregnancy: a clinical approach. *Obstetrics, Gynaecology and Reproductive Medicine* 2009; 19(1):19-25.

## Study design: Systematic or narrative review (n=3)

1. Burrell C, Edozien LC. Surrogacy in modern obstetric practice. *Seminars in Fetal and Neonatal Medicine* 2014; 19(5):272-278.
2. Conard J, Plu-Bureau Horellou M-H, Samama M-M, Gompel A. Thrombosis and assisted reproductive techniques (ART). *Journal des maladies vasculaires* 2011; 36(3):145-154.
3. Jacquesson L, Belaisch-Allart J, Ayel J-P. Induction of ovulation. *Journal de Gynecologie Obstetrique et Biologie de la Reproduction* 2010; 39 (8 SUPPL. 2): S67-S74.

## Study design: other (e.g., commentary, editorial) (n=5)

1. Ethics Committee of the American Society for Reproductive Medicine Ethics Committee of the American Society for Reproductive Medicine Provision of fertility services for women at increased risk of complications during fertility treatment or pregnancy: an Ethics Committee opinion. *Fertility and Sterility* 2016; 106(6):1319-1323.
2. American College of Obstetricians and Gynecologists' Committee on Ethics and Ryan, Ginny L. ACOG Committee Opinion No. 660: Family Building Through Gestational Surrogacy. *Obstetrics and Gynecology* 2016; 127(3):e97-e103.
3. Reddy UM, Wapner RJ, Rebar RW, Tasca RJ. Infertility, assisted reproductive technology, and adverse pregnancy outcomes: executive summary of a National Institute of Child Health and Human Development workshop. *Obstetrics and Gynecology* 2007; 109(4):967-977.
4. Takyi A, Santolaya-Forgas J. Prenatal screening for chromosomal abnormalities in IVF patients that opted for preimplantation genetic screening/diagnosis (PGS/D): a need for revised algorithms in the era of personalized medicine. *Journal of Assisted Reproduction and Genetics* 2017; 34(6):723-724.
5. Perinatal Risks Associated with IVF. RCOG, 2012.

<https://www.rcog.org.uk/globalassets/documents/guidelines/scientific-impact-papers/sip_8.pdf>
